# Engineering Dual-Loaded PLGA Nanoparticles with Gold Nanorods and Doxorubicin as Robust Multimodal Nanoplatforms

**DOI:** 10.1021/acsomega.5c10824

**Published:** 2026-02-06

**Authors:** İrem S. İlçi, Yağmur Zengin, Banu Iyisan

**Affiliations:** † Biofunctional Nanomaterials Design (BIND) Laboratory, Institute of Biomedical Engineering, 206540 Bogazici University, 34684 Istanbul, Turkey; ‡ Center for Targeted Therapy Technologies (CT3), 206540 Bogazici University, 34684 Istanbul, Turkey

## Abstract

Given the complexity of cancer, combination approaches such as chemo-photothermal and image-guided therapies are increasingly explored, driving interest in nanocarriers that integrate multiple structural abilities within a single platform. Here, we report a dual-functional nanoplatform in which gold nanorods (AuNRs) and doxorubicin (DOX) are coencapsulated in poly­(lactic-*co*-glycolic acid) (PLGA) nanoparticles (mean diameter ≈ 246 nm) after optimizing the amount of gold nanorods to be encapsulated. The dual-loaded nanoformulation yields a narrow size distribution (PDI ≤ 0.1), an adequate DOX level (32 ± 4 μg mL^–1^) for chemotherapeutic efficacy, and sufficient Au content (encapsulation efficiency of 48%) to achieve an enhanced temperature increase under NIR irradiation. Comprehensive stability testing for both AuNR-encapsulated PLGA Nps and bare-PLGA Nps was performed. Continuous centrifugation–redispersion cycles and 110-day storage at 4 °C reveal no measurable aggregation, shape deformation, or LSPR dampening in PLGA-encapsulated AuNRs, whereas free AuNRs lost their signal under the same conditions. Upon 808 nm irradiation (1 W cm^–2^, 5 min) the PLGA-Au-DOX nanospheres create ∼25 °C temperature difference, confirming intact photothermal performance. MTT assays in MCF-7 human breast cancer cells show that cytotoxicity is dominated by the chemotherapeutic payload in the designed system. The findings after detailed gold nanorod encapsulation optimization and stability studies indicate that the resulting nanoparticle is a well-characterized, dual-loaded, and reliable nanocarrier candidate for future in vivo studies on dual-modality cancer theranostics.

## Graphical abstract

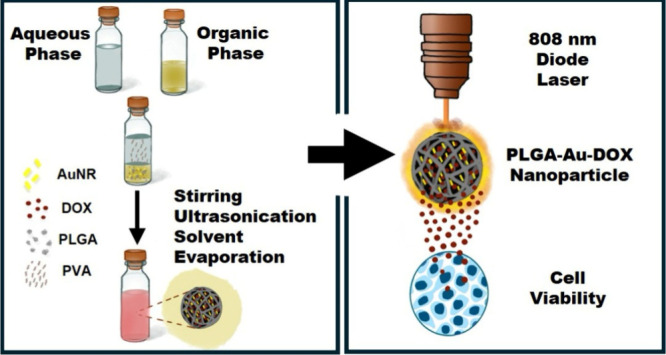

## 1 Introduction

The limitations of traditional anticancer drugs highlight the need for nanoparticle-based, externally triggered controlled drug delivery to tumors, thereby improving patient compliance and treatment outcomes.^1,2^ Combined strategies use various therapeutic approaches to target dominant tumor cell populations and drug-tolerant cells following treatment, resulting in long-term durable responses. For instance, radiotherapy, chemotherapy, or immunotherapy have been combined to decrease tumor development in preclinical investigations.^3^ In addition, image-guided drug delivery is also promising to enhance the success rate in cancer therapy.^4^ For this purpose, combining several functions in a single system is necessary and thus development of multimodal nanocarriers that show diverse abilities in one structure is gaining high interest.^5,6^

Light is a convenient tool as a rapid and clean external stimulus for the design of multimodal nanocarriers. One way of integrating light-sensitivity is to encapsulate gold nanorods, which convert light into heat^7–9^ and serve as versatile contrast agents for biomedical imaging techniques such as photoacoustic imaging.^10,11^ The first property can be used in photothermal therapy (PTT) to lead selective hyperthermia in a localized tumor area. Nanoparticles having high near-infrared (NIR) absorption penetrate deep tissue and heat locally, triggering cancer cell apoptosis, programmable cell death, while protecting neighboring tissues.^12^

The aspect ratio and size of gold nanorods (AuNRs) can be tuned and AuNRs with an aspect ratio greater than 3.5 exhibit longitudinal surface plasmon resonance (LSPR) peak within the optimal NIR absorption range (650–900 nm) for deeper-tissue penetration,^13^ enabling photothermal therapy alone or in combination with chemotherapy^14^ or cancer imaging.^15^ On the other hand, the toxicity of commonly used synthetic surfactants in gold nanorods synthesis remains a challenge, particularly when the nanoparticles are used alone.^16^ Therefore, integrating gold nanorods into a host nanocarrier system is promising not only for providing multimodal abilities but also for enhancing the biocompatibility of these effective metal nanoparticles. This approach can also benefit drugs such as doxorubicin (DOX), which is widely used in conventional cancer chemotherapy but whose systemic and particularly cardiac toxicity limit its clinical use.^17^ Therefore, to achieve high therapeutic efficacy while minimizing side effects, it is essential to develop a safe and effective multimodal nanoparticle based-delivery method for such therapeutic compounds.

Poly­(lactic-*co*-glycolic acid) (PLGA), which has FDA-approved formulations, is biocompatible and biodegradable, and provides the flexibility to control drug release kinetics with its varying lactide/glycolide ratio when used as a drug delivery vehicle.^18–21^ Because of these outstanding properties, it is a widely utilized material to form nanocarriers for use in biomedical applications. In particular, gold nanoparticles encapsulation into the PLGA nanocarrier offer clear advantages such as higher biocompatibility compared to bare gold nanoparticles,^22^ yet comprehensive stability data for these systems are still missing. Additionally, a few studies have focused on improving PLGA based nanostructures for combined applications such as chemo-photothermal therapy.^14,23^ However, the effective dual-encapsulation process to form multimodal systems remains challenging and requires detailed investigation. Existing studies often lack systematic data on optimizing dual encapsulation alongside long-term colloidal stability, hindering the advancement of both existing and new nanosystems for combination therapy or theranostics.

In this study, we present a structurally multimodal PLGA-based nanoplatform coencapsulating gold nanorods (AuNRs) and doxorubicin (DOX) synthesized via miniemulsion method, enabling both light-induced photothermal conversion and chemotherapeutic action under near-infrared (NIR) irradiation as illustrated in Figure 1. The nanoparticles were thoroughly characterized with respect to their physicochemical properties, AuNR loading dependent behavior, and long-term colloidal stability under various preparation and storage conditions. Laser studies focusing on NIR-triggered heating, drug-release profiles and cell viability on L929 mouse fibroblast cells and MCF7 breast cancer cells were investigated to evaluate the potential of the developed formulation as a dual-loaded nanocarrier platform.

**Figure 1:**
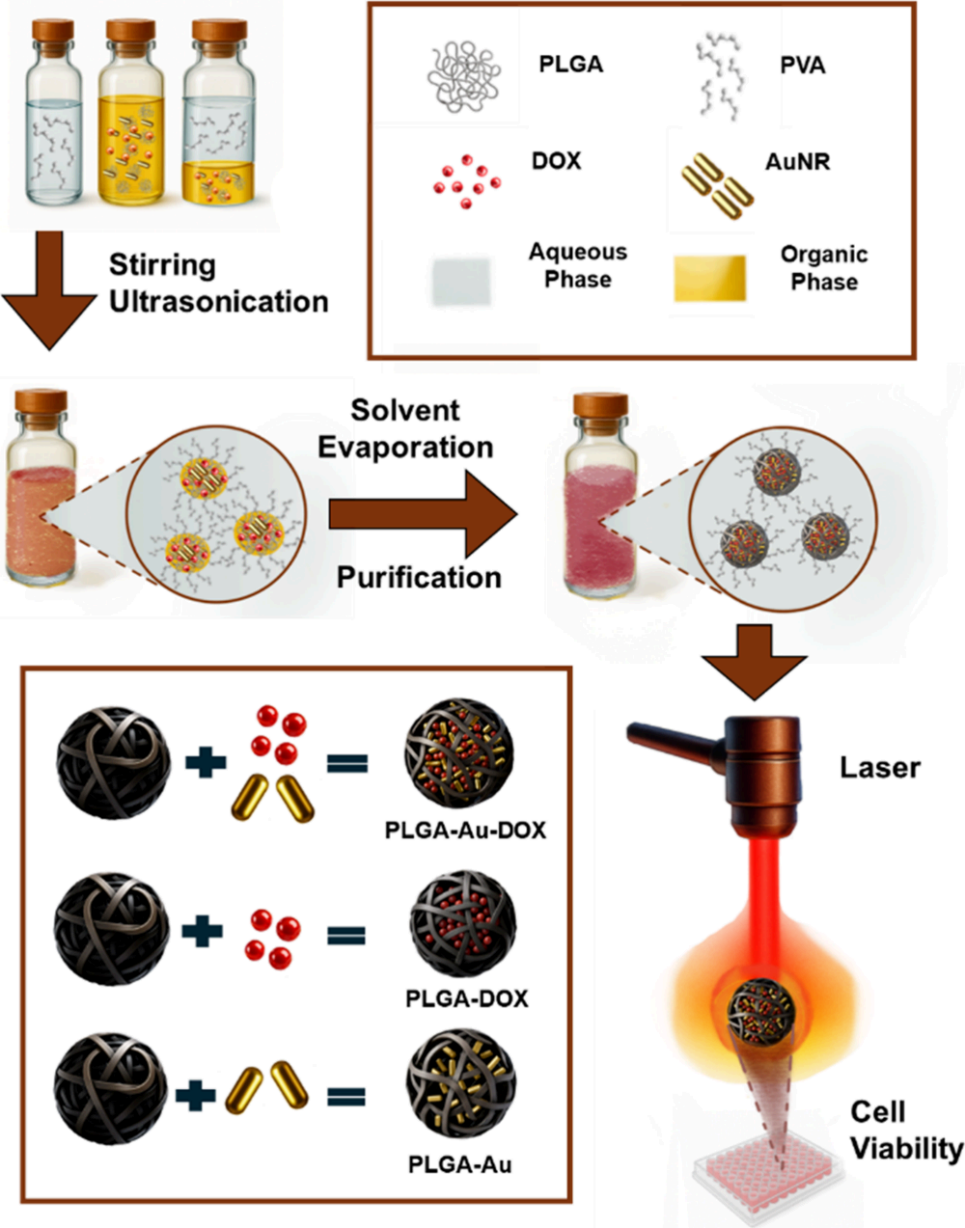
Schematic overview of this study, including the synthesis of PLGA nanoparticles containing gold nanorods (AuNRs) and/or doxorubicin (DOX), yielding PLGA-Au-DOX, PLGA-DOX, and PLGA-Au formulations, followed by NIR-laser treatment of MCF-7 cells.

## 2 Experimental Section

### 2.1 Materials

Poly­(lactic-*co*-glycolic acid) (PLGA, Resomer RG 503H; lactide:glycolide 50:50, acid-terminated, *M*_w_ 24–38 kDa) was purchased from Sigma-Aldrich. Chloroform (ACS reagent, ≥99.8%) was obtained from Fisher Scientific. Poly­(vinyl alcohol) (PVA; *M*_w_ 13–23 kDa, 87–89% hydrolyzed) was supplied by Sigma-Aldrich. Doxorubicin hydrochloride (DOX) was purchased from Biosynth Carbosynth. Dulbecco’s phosphate-buffered saline (DPBS) was obtained from Gibco, Thermo Fisher Scientific. Dialysis membrane (MWCO 6000–8000) was purchased from Carl Roth.

For cell culture studies Dulbecco’s Modified Eagle’s Medium (DMEM), fetal bovine serum (FBS, qualified origin from Brazil), Penicillin-Streptomycin (10,000 U/mL, 100×), Trypsin-EDTA (0.25% with phenol red) were acquired from Gibco; Dimethyl sulfoxide (DMSO, cell culture reagent) was sourced from NutriCulture; Thiazolyl Blue Tetrazolium Bromide (MTT Reagent, 98%) was obtained from Thermo Fisher; and Trypan Blue was purchased from Hyclone.

### 2.2 Preparation of PLGA Nanoparticles and Gold Nanorods

PLGA nanoparticles were produced using the miniemulsion technique, adapted from a previous method.^19^ The organic (dispersed) phase was prepared by dissolving 50 mg of PLGA in 2.5 mL of chloroform. In parallel, the aqueous phase was formed by dissolving 75 mg of poly­(vinyl alcohol) (PVA) in 10 mL of Milli-Q water with continuous stirring. The aqueous phase was slowly introduced to the organic phase while being magnetically stirred at 1000 rpm at ambient temperature (25 °C) for 1 h to create a macroemulsion. Subsequently, ultrasonication was conducted in an ice bath for 2 min utilizing a Branson Sonifier (450 W) set at 70% amplitude, employing 10 s on/10 s off cycles to produce a stable miniemulsion. The organic solvent was then evaporated by stirring the emulsion at 300 rpm for 16 h at ambient temperature, resulting in the creation of PLGA nanoparticles.

Gold nanorods (AuNRs) were synthesized using a seed-mediated growth technique as outlined in our previous study.^7^ Briefly, gold nano seeds were synthesized by reducing 0.01 M hydrogen tetrachloroaurate (HAuCl_4_·3H_2_O) solution with 0.01 M ice-cold sodium borohydride (NaBH_4_) in the presence of 0.1 M cetyltrimethylammonium bromide (CTAB). The mixture was gently stirred and subsequently incubated at 30 °C in a water bath. *CAUTION: HAuCl*_*4*_ *, a strongly acidic and oxidizing gold salt with corrosive and toxic properties, was handled in accordance with the material safety data sheet using appropriate personal protective equipment in a fume hood.* To prepare the growth solution, 0.01 M HAuCl_4_ was combined with 0.1 M CTAB. 0.1 M silver nitrate (AgNO_3_) and 0.1 M L­(+)-Ascorbic acid, respectively. Finally, the seed solution was incorporated into the growth solution. The resulting solution was maintained for 3 h in a 30 °C water bath. After 3 h, the solution was subjected to centrifugation at 4000 rpm and the solution was preserved at +4 °C.

### 2.3 Optimization of AuNR Loading

To determine the most suitable gold loading ratio for dual therapy applications, gold nanorods were encapsulated at varying mass ratios of 5, 10, 14, 27, and 41% with respect to a constant PLGA mass. The amount of AuNR corresponding to the relevant weight is determined according to the previously derived calibration curve (Figure S1) and the determined volume of gold nanorod was centrifuged at 12,000 rpm for 15 min. Briefly, PLGA was dissolved in chloroform and centrifuged AuNRs were added into vial and mixed at 1600 rpm for a minute to obtain the dispersed phase. Aqueous phase, containing PVA dissolved in Milli-Q water, was added dropwise into the dispersed phase under stirring at 1000 rpm. The macroemulsion was then formed by continuous stirring at 1000 rpm for 1 h at 25 °C. During the AuNR-loading optimization, samples were evaluated without centrifugation. Accordingly, STEM images obtained in this step correspond to noncentrifuged dispersions.

### 2.4 Dual Encapsulation of Gold Nanorods and Doxorubicin

Using the miniemulsion method, a final concentration of 1 mg mL^–1^ of Doxorubicin-HCl (DOX) and 0.714 mg/mL of gold nanorod encapsulated PLGA nanoparticles were synthesized. The quantity of AuNR equivalent to 7.14 mg has been determined as outlined in Section 2.3. Initially, DOX (10 mg) was dissolved in 0.3 mL of DMSO, followed by the addition of PLGA (50 mg) dissolved in chloroform (2.5 mL) and centrifuged AuNR at 12,000 rpm for 15 min were mixed at 1600 rpm for 1 min to achieve the dispersed phase. The subsequent steps of the synthesis were executed in accordance with the approach outlined in Section 2.2. Based on the optimal AuNR-loaded PLGA formulation identified in the previous section, both this formulation and the final samples were purified by centrifugation (2 × 30 min at 6000 rpm) and redispersed in Milli-Q water. Thus, STEM images reflect purified samples.

The content of encapsulated DOX was spectrophotometrically determined at 480 nm utilizing the Infinite M1000 microplate reader (Tecan, USA) according to the absorbance of collected supernatants during purification. The supernatants were diluted with dPBS in a 2:5 (sample:total) (v:v) ratio. The previously created DOX calibration curve was used to determine the concentration that corresponded to the absorbance value (Figure S2A).

The quantity of encapsulated gold nanorods (AuNRs) in the nanoparticle formulations was measured by thermogravimetric analysis (TGA). The measurements were performed in a nitrogen environment using TGA55 equipment (TA Instruments). In platinum pans, 1.036 mg of lyophilized PLGA-Au-DOX nanoparticles were heated to 800 °C at 10 °C/min. The residual mass at 600 °C was utilized to ascertain the gold concentration in the AuNR-loaded formulations. A defined volume of CTAB-stabilized gold nanorod dispersion was lyophilized, yielding a dried mass of 0.172 mg, which was analyzed under the same TGA conditions to determine the residual gold content. Encapsulation efficiency of AuNR based on TGA analysis was calculated as shown below:

$$\mathrm{EE}\%=\frac{\mathrm{Amount}\;\mathrm{of}\;\mathrm{encapsulated}\;\mathrm{Au}\;(\mathrm{mg})}{\mathrm{Amount}\;\mathrm{of}\;\mathrm{Au}\;\mathrm{initially}\;\mathrm{used}\;(\mathrm{mg})}$$

### 2.5 Characterization of Nanoparticles

The hydrodynamic diameter of the nanoparticles was determined by dynamic light scattering (DLS) using Zetasizer Lab (Malvern Instruments, U.K.) at a fixed scattering angle of 90° and reported as intensity-weighted average size (Z-average). For each measurement, 50 μL of nanoparticle suspension was diluted with 950 μL of Milli-Q water (1:20, v/v). Zeta potential (ζ-potential) measurements were also performed using the same instrument. Measurements were carried out in 1 mM KCl solution to maintain a standardized ionic environment.

The morphological characterization of the synthesized nanoparticles was performed via scanning transmission electron microscopy (STEM) using a Quattro S instrument (Thermo Fisher Scientific). For STEM analysis, diluted nanoparticle suspensions were dropped onto 300-mesh carbon-coated copper grids. Regarding AuNRs, the acquired STEM images were analyzed using ImageJ software to determine the length and width of individual nanorods.^24^ Dry-state diameters of PLGA-based nanoparticles were quantified from STEM images using an in-house MATLAB App (MATLAB R2024b, MathWorks). Particle segmentation was performed with an operator-adjustable threshold/sensitivity setting, and visually misdetected objects were excluded prior to calculating the final size distributions. Size data are reported as mean ± SD with the corresponding particle counts (*n*) indicated.

The optical properties of the synthesized gold nanorods were characterized using a TECAN Infinite M Nano microplate reader, which operates within an absorbance range of 230–1100 nm. FTIR analysis has been conducted by using IR Spirit-T spectrometer, Shimadzu.

### 2.6 Differential Scanning Calorimetry Analysis (DSC)

DSC analysis of PLGA polymer, PLGA nanoparticles, PLGA–DOX, and PLGA–Au–DOX was performed using a TA Instruments DSC Q2000 under a nitrogen atmosphere. In the first heating cycle, the samples were heated from −50 to 180 °C at 10 °C·min^–1^. This was followed by a cooling cycle from 180 to −40 °C at 50 °C·min^–1^. Finally, the samples were reheated from −40 to 180 °C at 10 °C·min^–1^ (second heating cycle). The second heating cycle was used for *T*_g_ analysis.

### 2.7 Scanning Electron Microscopy–Energy-Dispersive Spectroscopy (SEM-EDS) Analysis

To verify the presence of gold nanorods within PLGA Np, SEM-EDS analysis was conducted on PLGA-Au and PLGA-Au-Dox using a Quattro S instrument (Thermo Fisher Scientific). For each measurement, 20 μL of nanoparticle suspension was diluted with 980 μL of Milli-Q water (1:50, v/v). Seven μL of diluted nanoparticle suspensions were dropped onto 300-mesh carbon-coated copper grids for imaging and elemental analysis. Carbon (C), oxygen (O), and gold (Au) were elementally analyzed in 10 defined measurement points.

### 2.8 In Vitro Doxorubicin Release

The in vitro release profile of doxorubicin (DOX) was evaluated for both PLGA-DOX and PLGA-Au-DOX nanoparticle formulations using the dialysis method. To match the pH/ionic strength of the release environment prior to dialysis, the nanoparticle dispersions were redispersed in to dPBS (pH 7.4) after the last centrifugation step. The nanoparticle suspension (1 mL) was then introduced into the dialysis membranes with a molecular weight cutoff (MWCO) 6–8 kDa. Each formulation was tested in triplicate (*n* = 3). The dialysis bags were submerged in beakers containing 13 mL of Dulbecco’s Phosphate Buffered Saline (dPBS, pH 7.4). The release medium was maintained at 37 °C and stirred at 300 rpm using a magnetic stirrer with temperature control. Samples (600 μL) of the release medium were withdrawn at predetermined time points: hourly for the first 4 h, followed by approximately every 24 h until 96 h. Following each sampling, an equal volume (600 μL) of fresh dPBS was added to maintain sink conditions and constant volume.

The released DOX content in each sample was quantified by measuring absorbance at 480 nm using a UV–vis spectrophotometer (Infinite M Nano, TECAN, USA), after allowing the taken samples to reach room temperature. Each measurement was performed in triplicate (*n* = 3). DOX concentration was calculated using a previously established calibration curve (Figure S2A).

### 2.9 Stability Assessment of Nanoparticles

Two separate stability studies were designed to evaluate the behavior of nanoparticles under different medium and mechanical conditions. Each study was assigned a specific code for clarity and consistent reference throughout the manuscript. STB-Buffer (Stability-Buffer) focused on assessing the stability of PLGA nanoparticles in different buffer systems (sodium acetate, sodium phosphate, and phosphate-buffered saline) with varying pH values by monitoring them over 342 days through DLS analysis. On the other hand, STB-RPM (Stability-RPM) evaluated the impact of different centrifugation protocols on gold nanorod stability. PLGA nanoparticles and gold nanorods were subjected to sequential centrifugation steps with varying speeds, followed by storage for 110 days and monitoring through UV–vis and STEM analysis.

#### 2.9.1 STB-Buffer

Synthesized free PLGA nanoparticles were stored in three different buffer media (Phosphate-buffered saline at pH 7.4, sodium phosphate buffer at pH 6.5, and sodium acetate buffer at pH 5.5) at 4 °C and sampled at defined time intervals to evaluate the structural stability of the encapsulated systems.

2.5 mL of nanoparticle suspension was subjected to two sequential centrifugation cycles at 6000 rpm for 30 min after synthesizing the nanoparticle. After each cycle, the pellet was resuspended in 2.5 mL of fresh buffer and stored at 4 °C.

#### 2.9.2 STB-RPM

The STB-RPM study was designed based on the cumulative centrifugation steps to which gold nanorods (AuNRs) are exposed throughout their synthesis and subsequent processing in PLGA-Au nanoparticles. To this end, two different centrifugation protocols were designed and compared throughout the study. RPM1 includes centrifugation steps with an initial spin at 4000 rpm, which is typically applied during AuNR synthesis, followed by 12,000 rpm. It represents the condition of unencapsulated AuNRs just before encapsulation. In RPM2 condition, after the 4000 and 12,000 rpm steps, two additional centrifugation cycles at 6000 rpm were introduced. In RPM2 setting was performed on both free AuNRs and PLGA-Au formulations. RPM2 thus represents the final purified state of the formulations.

### 2.10 Photothermal Irradiation Studies

Transparent microcentrifuge tubes containing 0.3 mL of PLGA Np, gold nanorod (AuNR), PLGA-Au Np, and PLGA-Au-DOX Np dispersions were exposed to radiation for 300 s using a continuous-wave (CW) diode laser set to 808 nm with power densities of 0.5, 0.75, and 1 W cm^–2^. The content of AuNR was determined using the previously prepared calibration curve (Figure S1). All trials were conducted in triplicate (*n* = 3). Thermal camera (FLIR E5 XT Wifi) angled 90° with respect to the laser and the sample surface. The laser power density was validated with a calibrated power meter (Newport Model 1918-R). The laser point size was set to 0.5 cm^2^, guaranteeing complete coverage of the sample’s top surface. Thermal imaging was performed at 30 s intervals, and peak temperatures were ascertained with FLIR Tools software.

### 2.11 Cell Culture

MCF-7 human breast cancer cells (ATCC HTB-22) and L929 mouse fibroblast cells (ATCC CCL-1) were used in this study. Both cell lines were cultured in high-glucose Dulbecco’s Modified Eagle Medium (DMEM, Gibco), supplemented with 10% (v/v) fetal bovine serum (FBS, Gibco) and 1% penicillin/streptomycin (Gibco). Cells were maintained at 37 °C in a humidified incubator with 5% CO_2_ atmosphere (Nüve EC160, Turkey). Cells were removed for passage using 0.25% trypsin-EDTA (Gibco) for 5 min under regular incubation conditions. The enzymatic process was suppressed by the addition of full growth media, and the cell suspension was centrifuged at 1000 rpm for 5 min. The supernatant was removed, and the cell pellet was resuspended in fresh medium. Cell viability and density were evaluated by an automated cell counter (BIO-RAD TC20) using of trypan blue staining.

### 2.12 Cell Viability (MTT) Assay

Cytotoxicity was assessed in accordance with ISO 10993-5:2020 using L929 mouse fibroblast cells (ATCC CCL-1) as the reference cell line. Cell viability was evaluated by the 3-(4,5-dimethylthiazol-2-yl)-2,5-diphenyltetrazolium bromide (MTT) assay. Ten thousand cells per well were seeded into 96-well plates with 100 μL of complete growth media. Following a 24-h incubation period, the medium was substituted with new media containing nanoparticles at diverse final concentrations (10 to 120 μg mL^–1^), and the cells were then incubated for a further 24 or 48 h.

DMSO was utilized as the blank, while untreated cells cultured just with complete growth medium functioned as the negative control group. Following each incubation time, the medium was disposed of and 100 μL of PBS was used to gently wash the cells. Subsequently, 100 μL of fresh DMEM supplemented with 10 μL of MTT solution (5 mg mL^–1^) (MTT reagent, 98%, Thermo Fisher) was introduced to each well. Following a 4 h incubation in the incubator, the medium was meticulously extracted, and the resultant formazan crystals were solubilized in 100 μL of DMSO.

Absorbance was quantified at 570 nm with a Bio-Rad iMark microplate reader. There were six tests for each condition, and the mean ± standard deviation was used to represent the results.

### 2.13 Photothermal Cytotoxicity in MCF-7 Cells

To ensure identical conditions, including potential changes during irradiation, laser-exposed and control cells for each nanoparticle group were seeded on the same 96-well plate and underwent the same handling steps. Nanoparticles were added at predetermined final concentrations (150 and 250 μg mL^–1^) after a 24 h cell attachment period. To minimize thermal interference, one empty well was left between each sample well. After 24 h of nanoparticle incubation, the wells were washed with dPBS and replaced with fresh medium. Wells designated for photothermal treatment were irradiated with an 808 nm laser at 1 W cm^–2^ for 300 s, while the remaining wells were kept under the same external conditions without laser exposure. Plates were then incubated for 24 h to allow drug release from the polymer matrix, followed by MTT-based viability assessment.

Prior to selecting the laser parameters for photothermal treatment, control experiments were conducted to assess the effect of laser exposure alone on cell viability. MCF-7 cells seeded in 96-well plates (without nanoparticle treatment) were exposed to near-infrared (NIR) laser irradiation at various power densities and durations: 1 W cm^–2^ for 5 min, 1.7 W cm^–2^ for 5 min, and 2 W cm^–2^ for 10 min. Following irradiation, the cells were incubated for 24 h under standard culture conditions.

### 2.14 Statistical Analysis

Statistical analyses were performed using OriginPro 2025 (OriginLab Corporation, Northampton, MA, USA). For experiments in which nanoparticle-treated cells were subjected to laser irradiation (*n* = 3), a three-way ANOVA followed by Tukey’s post hoc test was applied, with *p* < 0.05 considered statistically significant. All results are presented as mean ± standard deviation (SD).

## 3 Results and Discussion

### 3.1 PLGA Nanoparticle Synthesis and Optimizing AuNR Encapsulation

PLGA nanoparticles were synthesized by the miniemulsion-solvent evaporation method as shown in Figure 1. Prior to AuNR encapsulation, blank PLGA nanoparticles (PLGA_initial_, Table 1) were synthesized following a protocol previously optimized in our laboratory.^19^ The continuous phase, containing PVA dissolved in ultrapure water, and the dispersed phase, consisting of PLGA dissolved in chloroform, were mixed under high shear stress to form a macroemulsion. Miniemulsion was then obtained by ultrasonication, followed by solvent evaporation for 16 h to remove chloroform. To ensure the complete removal of residual solvent, FTIR spectra of pure chloroform, PLGA nanoparticles before evaporation, and after evaporation were recorded, as shown in Figure S3. Chloroform exhibited distinct peaks at 751 cm^–1^ (C–Cl stretching) and 1218 cm^–1^ (C–H bending). These peaks were absent in the postevaporation PLGA nanoparticle spectrum, confirming the complete removal of chloroform during the synthesis.

**Table 1:** Hydrodynamic Size and Polydispersity Index (PDI) Values of PLGA Nanoparticles Encapsulating Gold Nanorods at Varying Loading Ratios^a^

sample Np	AuNR (% w/w of PLGA)	size (nm)	PDI	zeta potential (mV)
PLGA_initial_	0	220 ± 1	0.11 ± 0.01	–2.0 ± 0.7
PLGA-Au1	5	293 ± 8	0.09 ± 0.02	–1.4 ± 0.5
PLGA-Au2	10	287 ± 3	0.09 ± 0.10	–6.0 ± 0.2
PLGA-Au3	14	213 ± 1	0.10 ± 0.01	–1.0 ± 0.7
PLGA-Au4	27	297 ± 5	0.15 ± 0.03	–10.0 ± 0.2
PLGA-Au5	41	228 ± 3	0.30 ± 0.02	–4.0 ± 0.4

a Measurements were performed by dynamic light scattering (DLS) in aqueous media before purification.

The successfully synthesized acid-terminated PLGA nanoparticles served as a starting point and control for the AuNR- and/or doxorubicin-loaded formulations and were used to evaluate the structural stability of the system. The size of the blank PLGA nanoparticles was determined as 220 nm with a polydispersity index (PDI) of 0.11 by DLS measurements (Table 1), supporting the monodisperse and uniform size distribution. This initial characterization acted as an essential baseline for evaluating subsequent impact of AuNR loading on particle size, morphology, and stability. In the literature, the hydrodynamic diameter of blank PLGA nanoparticles produced by using PVA generally varies between ∼92–258 nm depending on the synthetic method.^19,25,26^ The nanoparticles developed in this study (∼220 nm) are positioned toward the upper end of this range, which is often associated with higher encapsulation capacity due to increased core volume, while remaining within the biologically suitable size window for effective delivery.

Prior to the fabrication of drug- and AuNR-dual-loaded nanoparticles, we conducted an AuNR encapsulation optimization study to identify the most suitable gold nanorod (AuNR) loading for effective photothermal performance. Five AuNR loaded-PLGA nanoparticle formulations (PLGA-Au1 to PLGA-Au5) were prepared by varying AuNR concentrations while keeping the PLGA content constant (Table 1). The selection of the optimal formulation was based on three key criteria: favorable physicochemical properties, uniform dispersion of AuNRs across the PLGA nanoparticles, and successful incorporation of AuNRs into the polymeric matrix. Visual inspection, STEM imaging, DLS measurements, and FTIR analysis collectively guided the identification of the most promising formulation for subsequent applications.

PLGA-Au1 and PLGA-Au2 Nps showed a radical increase in average hydrodynamic radius despite the relatively low amount of gold nanorod encapsulation compared to the blank nanoparticle (Table 1). According to the obtained STEM images, the PLGA-Au1 and PLGA-Au2 coded formulations did not exhibit a homogeneous distribution of gold nanorods among the nanoparticles and some free gold nanorods were observed outside the nanoparticles (Figure 2A,B). While the hydrodynamic radius of the PLGA-Au4 formulation, which added more gold nanorods than PLGA-Au3, was seen to be around 297 nm and had a PDI of 0.15, the size of the PLGA-Au3 coded sample seemed ideal as 210 nm and a PDI value of 0.1 (Table 1). Despite a higher gold nanorod loading rate, the PLGA-Au4 formulation was not considered suitable, as unencapsulated gold nanorods were more frequently observed in the STEM images. Conversely, PLGA-Au3 formulation demonstrated homogeneous AuNR distribution, favorable physicochemical properties, and successful encapsulation (Table[Table tbl1] and Figures 2C,F, S4). In the highest loading trial (PLGA-Au5, Figure 2E,F and Table 1), where gold nanorods were added at a rate of 41% by mass relative to PLGA, particle aggregation occurred at the end of the synthesis. The aggregated particles were removed from the main suspension by filtration and analyzed via Fourier-transform infrared spectroscopy FTIR (Figure S5). FTIR profiles of the raw PLGA and AuNR components indicate that the two materials interact upon mixing, resulting in aggregated PLGA–AuNR structures. The remaining dispersion, cleared of aggregated particles, was further examined by STEM imaging and DLS measurement. DLS results also showed a relatively high PDI value (0.3) due to this aggregation despite the particle size being well within the desired range. Therefore, this formulation was considered unsuitable for further applications and was excluded from further experiments due to its aggregation-prone nature.

**Figure 2:**
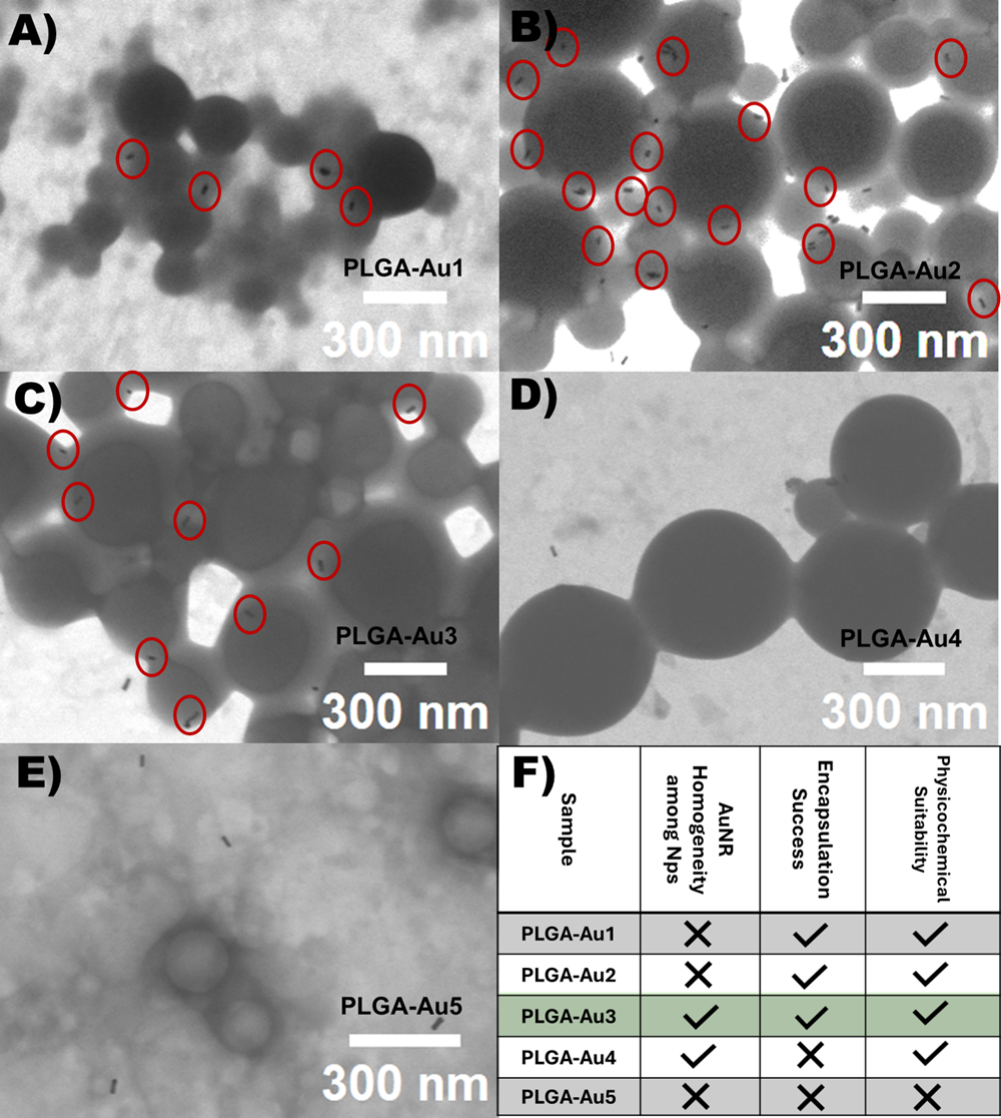
STEM images of PLGA nanoparticles encapsulating gold nanorods at varying loading ratios. (A) PLGA-Au1, (B) PLGA-Au2, (C) PLGA-Au3 (selected as optimal formulation), (D) PLGA-Au4, and (E) PLGA-Au5. (F) Comparative summary for selection of the optimal formulation.

Based on encapsulation efficiency, PLGA-Au3 Np was the best gold nanorod (AuNR)-loaded nanoparticle system in the present study. After evaluating the optimal AuNR loading capacity, purification was performed since analyzing encapsulation before purification better represents the initial encapsulation state. Optimum formulation (PLGA-Au3 Np) was centrifuged to become PLGA-Au Np, the optimized system’s pure form. Purification was necessary to remove toxic, CTAB-coated, and nonencapsulated gold nanorods (AuNRs) from the emulsion. The nanoparticle system would also contain doxorubicin (DOX) later in the trial. After the second encapsulation stage, unencapsulated DOX molecules and AuNRs had to be removed to improve drug loading and experimental accuracy.

### 3.2 Dual Encapsulation and Drug Release Performance

After DLS and STEM analyses, the PLGA-Au3 Np sample, selected as the optimum formulation, was purified in two centrifugation steps of 30 min each at 6000 rpm, and this purified nanoparticle was referred to as PLGA-Au Np. The hydrodynamic radius of the PLGA-Au Np increased slightly upon centrifugation, from approximately 213 to 221 nm, and the PDI value decreased from 0.1 to 0.06. For proper comparison, the PLGA_initial_ nanoparticles listed in Table 1 were subjected to the same centrifugation parameters, and characterizations were repeated after this purification step (PLGA_purified_). Even after the excessive centrifugation steps, PLGA and PLGA-Au nanoparticles showed intact structure and monodisperse behavior as supported by DLS measurements (Table 2) and STEM imaging (Figure 3A,B). In our case, the purified and selected optimum formulation (PLGA-Au Np) exhibited a mean diameter of approximately 220 nm (Table 2) after centrifugation, indicating a relatively smaller and more compact particle size compared to previous reports. The diameters of PLGA nanoparticles incorporating gold nanorods, as reported in the literature, range from approximately 350 to 500 nm, depending on the specific formulation and production method employed.^27,28^

**Table 2:** Hydrodynamic Size and Polydispersity Index (PDI) Values of Different Nanoparticle Formulations: Blank PLGA Nanoparticles, PLGA-DOX Nanoparticles, PLGA Nanoparticles Encapsulating Gold Nanorods (PLGA-Au), and PLGA Nanoparticles Co-Loaded with Gold Nanorods and Doxorubicin (PLGA-Au-DOX)^a^

sample	size (nm)	PDI	zeta potential (mV)
PLGA_purified_Np	229 ± 1	0.09 ± 0.02	–1.4 ± 0.5
PLGA-Au Np^b^	221 ± 2	0.06 ± 0.02	–2.4 ± 0.9
PLGA-DOX Np	241 ± 4	0.08 ± 0.02	–15.6 ± 1.3
PLGA-Au-DOX Np	246 ± 3	0.09 ± 0.02	–16.3 ± 1.2

a Measurements were obtained by dynamic light scattering (DLS) after purification.

b PLGA-Au Np refers to the purified form of the optimally selected formulation, which is designated as PLGA-Au3 in Table 1 *.*

**Figure 3:**
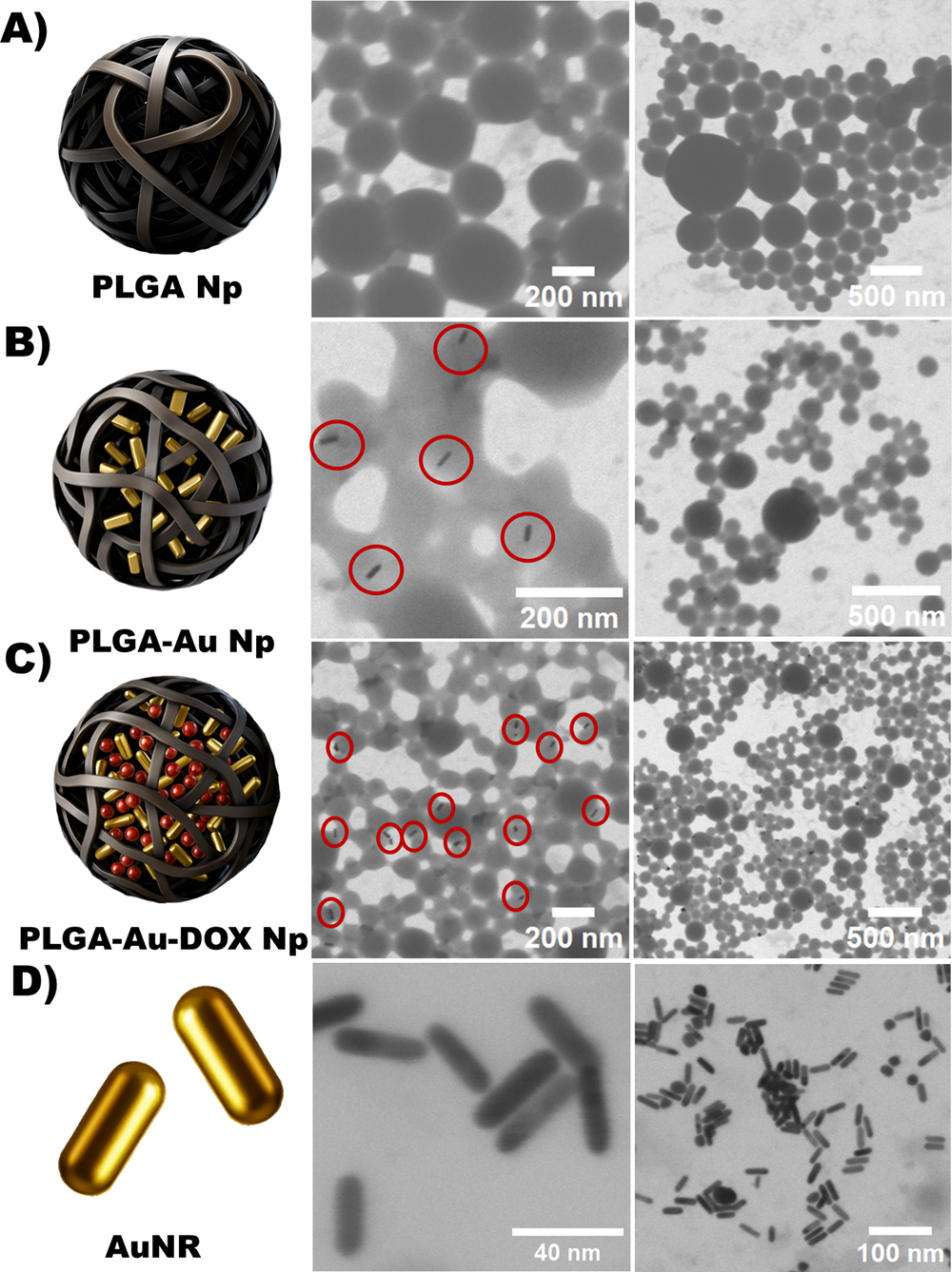
Scanning transmission electron microscopy (STEM) images of (A) blank PLGA nanoparticles, (B) PLGA-Au nanoparticles loaded with gold nanorods (AuNRs), (C) PLGA-Au-DOX nanoparticles coloaded with AuNRs and doxorubicin (DOX), and (D) AuNRs. Each row presents representative images at different magnifications, confirming the morphology and successful encapsulation of the gold nanorods.

After the successful integration of gold nanorods (AuNRs) into PLGA nanoparticles and the selection of purification parameters (6000 rpm, 30 min, repeated twice), doxorubicin (DOX) was subsequently encapsulated into the nanoparticle system. The hydrodynamic diameter of these DOX-encapsulated PLGA nanoparticles is relatively larger (∼241 nm) than that of blank PLGA and PLGA-Au Np and similar to that of PLGA-Au-DOX Np (∼246 nm). These slightly increased particle sizes compared to blank PLGA nanoparticles, related to the inclusion of gold nanorods and doxorubicin. Nonetheless, their size distributions remained within the nanoscale range, with PDI values below 0.1, indicating monodispersity and formulation uniformity. Zeta potential measurements revealed negative surface charges close to zero for all formulations, reflecting the nonionic surfactant, PVA, used during synthesis. Despite the low surface charge, colloidal stability was likely maintained through steric stabilization provided by the adsorbed nonionic surfactant as consistent with the previous studies.^19,29,30^ DLS results report the hydrodynamic diameter in water (220–250 nm), whereas STEM shows the particles in the dry state, where they can appear smaller. To quantify this, we performed a statistical size analysis from STEM images (*n* = 150) and obtained dry-state diameters of 174 ± 17 nm (PLGA NP), 117 ± 3 nm (PLGA–Au NP), and 100 ± 6 nm (PLGA–Au–DOX NP) (Figure S6). Drying-induced shrinkage is commonly observed in nanoparticle systems and explains the difference between the two measurements.

In the STEM images of PLGA-Au and PLGA-Au-DOX nanoparticles, the gold nanorods (highlighted with red circles) are clearly embedded within the polymer matrix while retaining their original morphology (Figure 3B,C). The persistent spherical structure of the PLGA nanoparticles further suggests that AuNRs were successfully incorporated without compromising particle integrity. Moreover, the bare AuNRs imaged prior to nanoparticle synthesis (Figure 3D) maintained consistent dimensions and rod-like morphology throughout the process, confirming their structural stability and suitability for photothermal applications. In addition to STEM imaging, Scanning Electron Microscopy–Energy Dispersive Spectroscopy (SEM-EDS) analysis was performed on PLGA-Au and PLGA-Au-Dox nanoparticles to confirm the presence of gold nanorods within the PLGA Np. As shown in Figure S7, nanoparticles were examined for their elemental composition, specifically carbon (C), oxygen (O), and gold (Au). The detection of Au element verifies the incorporation of gold nanorods, while the presence of C and O originates from the PLGA polymer, the primary structural component of the nanoparticles. Gold signals were observed at the majority of the defined measurement points on the PLGA-Au and PLGA-Au-DOX, verifying its presence.

On the other hand, UV–vis and FTIR analysis were also conducted to support the encapsulation of gold nanorods and doxorubicin into PLGA nanoparticles. The UV–vis spectra of PLGA Np, PLGA-Au Np, PLGA-DOX Np, PLGA-Au-DOX Np, AuNR, and DOX were obtained as given in Figure S8. As can be seen in Figure S8A, the characteristic LSPR peak of gold nanorods in NIR shown in Figure S8B created a shoulder in both PLGA–Au and PLGA–Au–DOX nanoparticles as an indication of AuNR. Additionally, a distinct absorbance increase around 480 nm which corresponds to the characteristic absorption of DOX shown in Figure S8C is evident in PLGA–DOX and PLGA–Au–DOX nanoparticles. The simultaneous presence of both DOX- and AuNR-related peaks in the PLGA–Au–DOX spectrum confirms the successful dual encapsulation of DOX and AuNRs within the PLGA Np. As seen in Figure S9, the FTIR spectrum of blank PLGA nanoparticles exhibited the characteristic ester C=O stretching at 1750 cm^–1^ and C–O–C stretching bands between 1080 and 1200 cm^–1^. CTAB-stabilized AuNRs showed intense aliphatic C–H stretching bands around 2990 and 2850 cm^–1^, confirming the presence of the surfactant on the nanorod surface. Free DOX displayed bands associated aromatic C=C/C=O vibrations (1600–1650 cm^–1^). In PLGA-Au-DOX nanoparticles, the ester C=O band of PLGA was preserved without significant shift, whereas the DOX-related signals appeared as broadened, low-intensity shoulders, and no additional peaks corresponding to crystalline DOX were detected. These findings are consistent with DOX being molecularly dispersed within the PLGA matrix rather than forming a separate crystalline phase on the nanoparticle surface.

In our previous study, we optimized the aspect ratio of gold nanorods to achieve the desired LSPR peak in the NIR region.^7^ Numerous studies have demonstrated that increasing the aspect ratio causes a red-shift of the LSPR peak toward the near-infrared (NIR) biological window, where light absorption by tissues and water is minimal, enabling deeper tissue penetration.^7,31,32^ AuNRs with an aspect ratio greater than 3.5 typically exhibit an LSPR peak in the NIR region, which matches the irradiation wavelength used for photothermal therapy in combination with chemotherapy.

Beyond their optical behavior, the biological fate of AuNRs after fulfilling their therapeutic function is also an important consideration. When gold nanorod-loaded PLGA nanoparticles are introduced into the body, PLGA degradation will lead to the gradual release of the gold nanorods. From an ADME perspective, intravenously administered gold nanorods enter systemic circulation and are distributed through the bloodstream. Following distribution, AuNRs typically accumulate in reticuloendothelial system organs, particularly in liver and spleen.^33^ Their biodistribution and clearance are strongly influenced by physicochemical properties such as size and shape. For instance, Talamini et al. reported that rod-shaped gold nanoparticles (60 × 30 nm) exhibited lower tissue penetration and more rapid renal clearance compared to gold nanostars and gold nanospheres.^34^

Thermogravimetric analysis (TGA) was conducted to evaluate the thermal stability and gold content of the PLGA-Au-DOX nanoparticle formulation (Figure S10). TGA analysis confirmed the gold content in the AuNR sample by leaving approximately 10.76% residue in CTAB-coated gold nanorods (AuNR). This result is in good agreement with the residue rate of around 12% reported in the literature for AuNRs with similar structure.^35^ This indicates that synthesized AuNRs have a large amount of CTAB and due to its cytotoxic nature, surface modification^27^ or encapsulation into biocompatible structures such as PLGA would be beneficial for biomedical applications as performed in this study. According to TGA results of PLGA-Au-DOX Np, 1.9% residue remained after thermal degradation. As shown in the TGA curve (Figure S10), the sample coded PLGA-Au-DOX Np exhibited an initial minor weight loss (∼0.88%) below 200 °C, attributed to moisture evaporation. The main degradation phase occurred between 250–450 °C, corresponding to the breakdown of the PLGA, PVA and encapsulated doxorubicin. The final residue at 800 °C was 1.9%, indicating the presence of thermally stable gold. The total amount of encapsulated pure gold in PLGA-Au-DOX was 0.66 mg with an encapsulation efficiency of 48% relative to the initial 1.37 mg pure gold introduced. In the literature, final residue was found to be 1.1% as a result of TGA analysis by Amirishoar et al. where gold nanoparticles-entrapped and folic acid-functionalized PLGA nanoparticles were fabricated.^36^ In this study, thermogravimetric analysis revealed that our formulation contained 1.8-fold higher gold content (in mg) compared to the formulation reported in one of the few previous studies.^36^ There is limited data in the literature on the quantification of gold content in such systems, and our formulation appears to exhibit a comparatively higher loading.

Differential scanning calorimetry (DSC) confirmed the amorphous nature of PLGA and all nanoparticle formulations (Figure S11). PLGA polymer showed a *T*_g_ of 45.14 °C with no detectable Tm or Tc peaks. PLGA nanoparticles exhibited a slightly reduced *T*_g_ (43.14 °C), consistent with increased chain mobility at the nanoscale. DOX-loaded PLGA nanoparticles showed a markedly lower *T*_g_ (28.25 °C), indicating a pronounced plasticizing effect of the drug.^37^ In contrast, AuNR and DOX-loaded nanoparticles displayed an intermediate *T*_g_ (37.87 °C), reflecting the opposing influences of DOX-induced plasticization and AuNR-induced chain restriction. The absence of melting and crystallization transitions in all samples confirms their predominantly amorphous character.

Doxorubicin (DOX) loading was quantified for both PLGA-DOX and PLGA-Au-DOX nanoparticle formulations. For each system, three independent batches were prepared and analyzed (*n* = 3). The PLGA-DOX formulations showed loaded DOX concentrations of 85 ± 22 μg mL^–1^. In comparison, the PLGA-Au-DOX formulations exhibited lower DOX concentrations, measured as 32 ± 4 μg mL^–1^. These results suggest that the inclusion of gold nanorods within the polymer matrix may reduce the drug loading capacity, potentially due to competitive encapsulation space during the formulation process. Similarly, a decrease in DOX loading efficiency during coencapsulation of AuNRs into human serum albumin (HSA) coated PLGA Nps has also been reported previously.^1^

The in vitro drug release (pH 7.4 at 37 °C) characteristics of PLGA-DOX and PLGA-Au-DOX nanoparticles exhibited an early burst phase, succeeded by a prolonged release plateau and subsequent steady-state phase (Figure S2). This result is in agreement with the previous studies of PLGA nanoparticles since their burst release profile is a known fact.^38^ Both formulations released a portion of the encapsulated doxorubicin during the initial hour, with negligible variation noted subsequently. This rapid release may be attributed to surface-adsorbed drug or weakly bound DOX near the polymer interface. This profile is completely consistent with the behavior reported in the literature, particularly for acid-terminated PLGA.^39,40^ In addition, although PLGA-DOX carried more drug than PLGA-Au-DOX, the amount of drug released by both was the same, implying a faster release from PLGA-Au-DOX. This behavior may be related to a formulation-dependent internal localization of DOX. Electrostatic attraction between the positively charged CTAB-AuNRs (+34.8 ± 2.2 mV) and negatively charged DOX (−15.1 ± 3.6 mV) likely promotes interaction, and the relatively higher hydrophilicity of these interacting payloads may favor their localization near the particle periphery during synthesis. Such a spatial arrangement would shorten the diffusion path and could account for the accelerated release kinetics. The proposed explanations for the reduced encapsulation and accelerated release, however, are inferred from formulation behavior, as the underlying interactions were not directly examined in this study.

### 3.3 Colloidal Stability of AuNR and PLGA Nanoparticles

To facilitate clinical translation, the long-term stability of the developed nanoparticles was first evaluated over 110 days under two centrifugation protocols mimicking storage and handling conditions. This investigation focused on how cumulative mechanical stress from multiple centrifugation steps influences the structural and optical stability of AuNRs, both in their bare form and when encapsulated within PLGA matrices. In contrast to prior reports, this work presents one of the few long-term, rpm-controlled, multistep stability evaluations of PLGA-Au nanospheres. Over 110 days, our protocol uniquely demonstrates that the encapsulated AuNRs preserve their morphology and optical properties for months, whereas bare AuNRs rapidly aggregate and lose their LSPR under identical conditions (Figure S12).

From the AuNR perspective, the process comprised sequential centrifugation at 4000 rpm, 12,000 rpm, and two consecutive spins at 6000 rpm. For loaded PLGA nanoparticles, only the postencapsulation purification step, two consecutive 6000 rpm centrifugations, was applied. In this context, RPM1 refers to PLGA-Au3 nanoparticles that were not subjected to this purification step (Table 1), while RPM2 refers to those that were purified (Table 2, PLGA-Au Np).

The longitudinal LSPR band of free AuNRs entirely disappeared under repeated centrifugation (RPM1, after 110 days of storage; RPM2, *t* = 0 and after 110 days of storage) (Figure S12B), coinciding with black precipitate formation and aggregates that are clearly visible by STEM imaging. STEM revealed 80–300 nm gold structures indicating advanced rod–rod fusion following CTAB loss (Figure S12A), consistent with literature reports.^41,42^ The loss of the longitudinal LSPR band reflects both shape/aggregation changes and the gradual reduction of nanorods during washing, which lowers the optical density in RPM2 setting. Conversely, AuNRs encapsulated in PLGA Nps predominantly preserved their rod morphology and optical signature even after 110 days under identical centrifugation conditions (Figures 4 and S12C). As shown in Figure S13 and Table S1, the length, width, and aspect ratio (AR) of gold nanorods at day 0 before encapsulation and gold nanorods encapsulated in PLGA Nps at day 0 and day 110 under RPM1 and RPM2 conditions were analyzed from STEM images using ImageJ. Before encapsulation, the aspect ratio (AR) of the gold nanorods was 3.7 ± 0.5. After encapsulation into PLGA Nps, the gold nanorods maintained AR values over time under both RPM1 and RPM2 conditions. Specifically, nanorods subjected to RPM1 conditions exhibited AR values of 3.2 ± 0.3 at both day 0 and day 110, while those under RPM2 conditions showed AR values of 3.6 ± 0.5 at day 0 and 3.3 ± 0.6 at day 110. For the nonencapsulated AuNRs, STEM imaging revealed mainly aggregated/cluster-like structures with no individually resolved rods in the scanned regions under RPM2 (*t* = 0) and under both RPM1 and RPM2 after 110 days of storage (Figure S12A). Therefore, aspect-ratio analysis for these samples was not performed. Under the analyzed experimental conditions, these results suggest that AuNR shape and colloidal integrity are retained across multiple washing and redispersion cycles when embedded in PLGA matrices, likely due to reduced CTAB loss.

**Figure 4:**
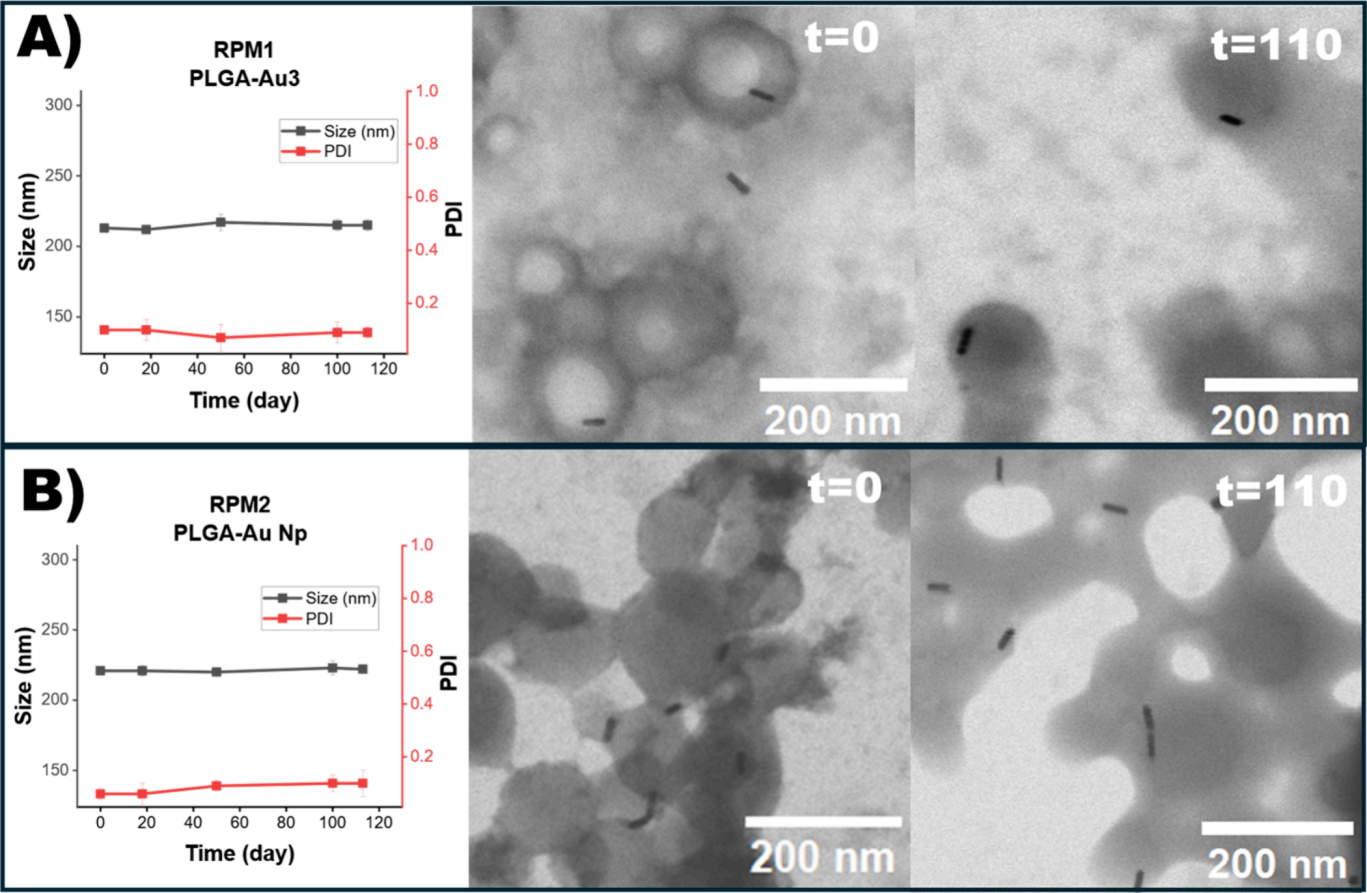
Stability analysis of gold nanorod-encapsulated PLGA nanoparticles subjected to different centrifugation protocols: (A) RPM1 and (B) RPM2.

For each condition, hydrodynamic size and polydispersity index (PDI) were monitored over a 110-day storage period by dynamic light scattering (DLS), and morphological changes were visualized using STEM imaging at day 0 (*t* = 0) and day 110 (*t* = 110).

Second, to evaluate the long-term colloidal and structural stability of PLGA nanoparticles under physiologically relevant conditions, samples were stored in buffer solutions at pH 7.4, 6.5, and 5.5 at 4 °C and monitored for over 300 days (Figures S14 and S15). The aim was to investigate how different ionic and pH environments influence the colloidal properties of the nanoparticles over time after removing some portion of surfactant through centrifugation and redispersion into corresponding buffers for stability testing. This study also provides important insight into how the PLGA nanoparticles, which constitute the core structure of our system, behave in different buffer conditions as a final product. Understanding this behavior is particularly valuable for anticipating the system’s performance in future applications.

In this study (STB-Buffer), nanoparticles were subjected to two centrifugation cycles to remove free surfactants before redispersion into corresponding buffers. The hydrodynamic diameter and polydispersity index (PDI) values of PLGA nanoparticles stayed quite consistent over the entire storage time, even when the pH conditions changed (Figures S14 and S15). In PBS (pH 7.4), the hydrodynamic diameter/PDI changed minimally from 279 nm/0.03 to 299 nm/0.15; in sodium phosphate buffer (pH 6.5), from 280 nm/0.05 to 287 nm/0.06; and in sodium acetate buffer (pH 5.5), from 289 nm/0.07 to 294 nm/0.09 over 342 days. This consistent stability across many settings indicates that the polymer matrix effectively maintained particle integrity, even in mildly acidic environments (pH 5.5 and 6.5), relevant to tumor microenvironments or endosomal conditions. Also, the similar size and PDI of centrifuged nanoparticles suggests that centrifugation removes only free surfactants (PVA), while the remaining PVA is tightly attached to the surface, contributing to the long-term colloidal stability of PLGA Nps. These findings illustrate the resilience of the miniemulsion synthesis technique and highlight the nanoparticles’ suitability for biomedical applications necessitating long-term stability, regardless purifying processes.

### 3.4 Laser Studies and Photothermal Response

Following the optimization of gold-nanorod and doxorubicin encapsulation, laser-irradiation studies were conducted to evaluate the photothermal conversion efficiency of the optimized formulation. A continuous-wave diode laser with a wavelength of 808 nm was employed, corresponding to the longitudinal localized surface plasmon resonance (LSPR) of the AuNRs with an aspect ratio above 3.5, positioning their LSPR peak within the near-infrared (NIR) biological window. The 808 nm wavelength was selected for its strong absorption by AuNRs and deep tissue penetration, enabling effective photothermal heating. For this purpose, the temperature changes caused by the heat generated by the synthesized PLGA-Au Nps and PLGA-Au-DOX Nps at different power densities were compared. Blank PLGA NPs were used as a control group to determine the heat changes caused by the AuNR presence. 0.3 mL of the synthesized samples were irradiated in centrifuge tubes, and the temperature changes were monitored with a thermal camera as illustrated in Figure 5D. The temperature elevation (Δ*T*) is used as a functional indicator to compare photothermal responses. As seen in the figure, the data indicate that encapsulating gold nanorods within PLGA produces controllable heat. The PLGA-Au-DOX nanoparticles induced a temperature increase of approximately 25 °C under NIR laser irradiation within 5 min, whereas blank particles remained almost constant at 1 W cm^–2^. In short, the increasing temperature trend with increasing power density was observed for PLGA-Au Np and PLGA-Au-DOX Np, whereas for PLGA Np without gold nanorods, the temperature remained stable at all three power densities. Amirishoar et al. reported a 10 °C rise after 60 min at 1 W cm^–2^, whereas our system achieved ∼25 °C in 5 min under the same conditions, indicating more efficient heat generation likely associated with the higher AuNR loading amount discussed earlier.^37^

**Figure 5:**
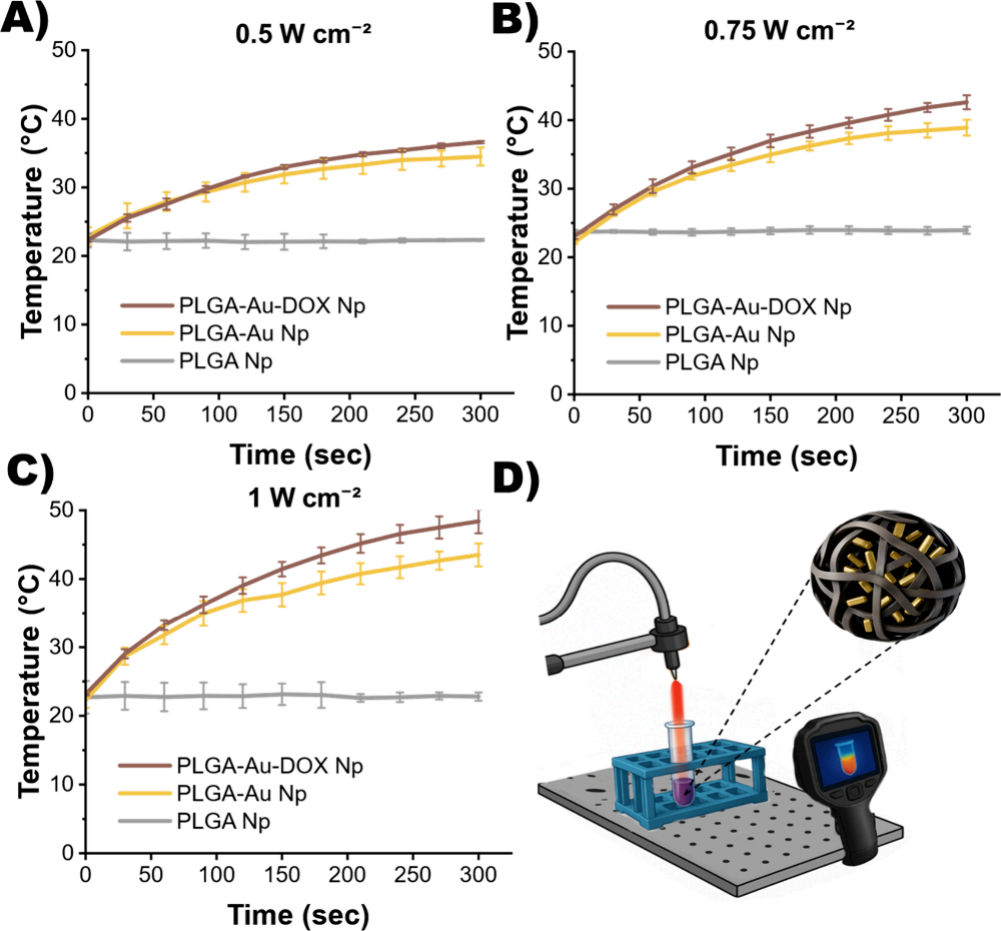
Photothermal response of PLGA-Au, PLGA-Au-DOX, and blank PLGA (control) nanoparticles at constant volume under different laser power densities: (A) 0.5 W cm^–2^, (B) 0.75 W cm^–2^, and (C) 1.0 W cm^–2^. (D) All measurements were performed using the illustrated laser setup.

In Table 3 where the temperature changes are shown, the temperature change obtained for PLGA Np at all three power densities are neglectable as a control group. The DOX-containing formulation consistently generates around 3–4 °C more heat than the Au-only equivalent under identical laser intensity, suggesting that DOX encapsulation does not compromise AuNR loading and the overall photothermal response. This minor variation, though within the range of experimental standard deviation, may reflect a subtle structural reconfiguration during dual-encapsulation where a higher fraction of AuNRs localizes near the particle periphery, thereby enhancing heat transfer to the surrounding medium. Apart from all these, the temperature change in gold-containing formulations is proportional to the applied power density. According to ANSI Z136.1 and ICNIRP safety standards, the commonly cited maximum permissible exposure (MPE) for continuous wave lasers on human skin is approximately 0.33 W cm^–2^ for an 808 nm laser.^43,44^ In our study, laser irradiation applied at three different power densities 0.5, 0.75, and 1 W cm^–2^ for 5 min. Although these values exceed the nominal MPE, the actual exposure also depends on other factors such as exposure time. Therefore, initial in vitro experiments are required to assess photothermal performance and resulting temperature increases. Several published studies have employed higher 808 nm laser power densities for in vitro photothermal experiments using gold nanorods. For instance, Ye et al.^45^ applied 0.5–2 W·cm^–2^, and Shan et al.^46^ reported photothermal testing at 2 W·cm^–2^. In comparison, maximum power density used in our study (1 W·cm^–2^) is lower than upper limits typically reported. In future perspective, similar heating performance can be achieved with lower power densities by adjusting nanoparticle loading concentration, irradiation duration, spot size, and targeting ability.

**Table 3:** Temperature Change of Different Formulations at Three Applied Power Densities after Five Minutes

sample	Δ*T* (°C) 0.5 W cm^–2^	Δ*T* (°C) 0.75 W cm^–2^	Δ*T* (°C) at 1 W cm^–2^
PLGA-Au Np	11.60 ± 0.70	16.70 ± 0.90	21.20 ± 1.60
PLGA-Au-DOX Np	14.30 ± 0.10	19.60 ± 1.30	25.30 ± 1.70

### 3.5 In Vitro Cytotoxicity and Photothermal Efficacy

Following the confirmation of favorable physicochemical properties after encapsulation, stability tests and laser studies, further investigations were carried out at the cellular level. The MTT cell viability assay for L929 mouse fibroblast cells (ATCC CCL-1) containing both empty PLGA and PLGA-Au nanoparticles, was conducted as per ISO 10993-5:2020 standards (Figure 6.

**Figure 6:**
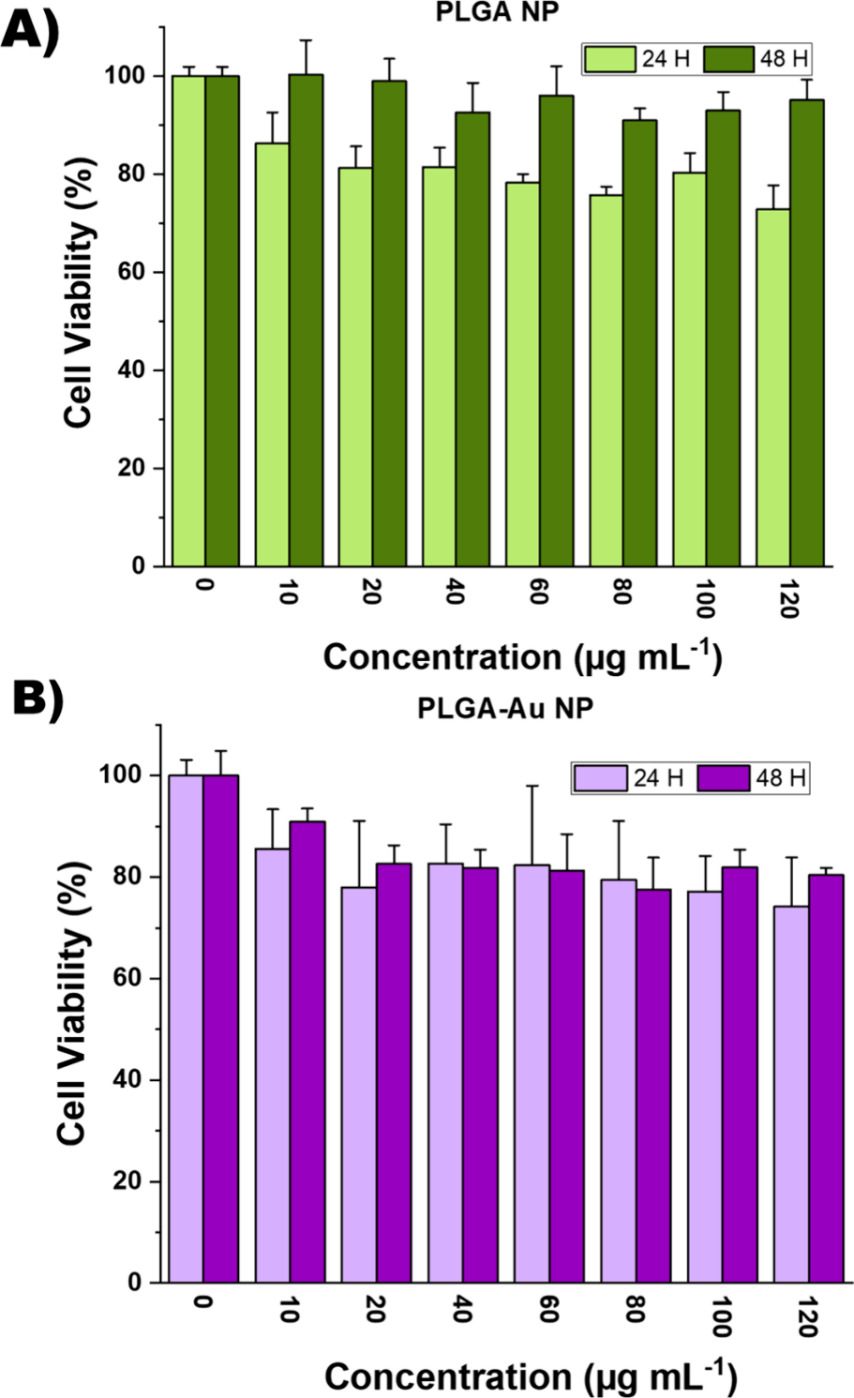
Cellular viability of (A) blank (PLGA Np) and (B) gold nanorod-encapsulated PLGA Np (PLGA-Au Np) in L929 mouse fibroblast cell line (*n* = 6).

Both PLGA and PLGA–Au NPs demonstrated favorable cytocompatibility toward L929 cells within the tested concentration range (0–120 μg mL^–1^). After 48 h exposure, PLGA NPs preserved near-complete viability (≥91%), confirming their biocompatibility. PLGA–Au NPs also maintained high viability (77–91%), indicating no severe cytotoxicity even at the maximum tested concentration.

At 24 h, both nanoparticle formulations caused a moderate reduction in cell viability, with values ranging from 72–86%. The effect was more pronounced at higher concentrations, particularly for PLGA–Au NPs, but viability consistently remained above 70%, suggesting only a transient stress response. Importantly, this reduction was not sustained, as cells exposed for 48 h exhibited recovery of viability, especially in the case of PLGA NPs, which returned to near-control levels. These results collectively indicate that PLGA NPs are highly cytocompatible, while PLGA–Au NPs also exhibit acceptable safety, with cell viability consistently remaining above the 70% biocompatibility threshold at all tested concentrations.

In order to determine the optimum laser conditions that do not affect cell viability, cells alone were exposed to laser irradiation at different power densities and exposure times (Figure 7A,B) prior to the laser treatment of nanoparticle-loaded cells. Irradiation at 1 W cm^–2^ for 5 min produced no change in cell viability compared to untreated cells, whereas increasing the power to 1.7 W cm^–2^ (5 min) reduced viability by 18 ± 8%. A further increase in both laser power and exposure time at (2 W cm^–2^ for 10 min) resulted in similarly reduced cell viability (83 ± 7%). On the basis of these data, the laser condition of 1 W cm^–2^ for 5 min was selected for subsequent experiments, as it produced the smallest deviation from the control group’s cell viability consistent with literature.^47^

**Figure 7:**
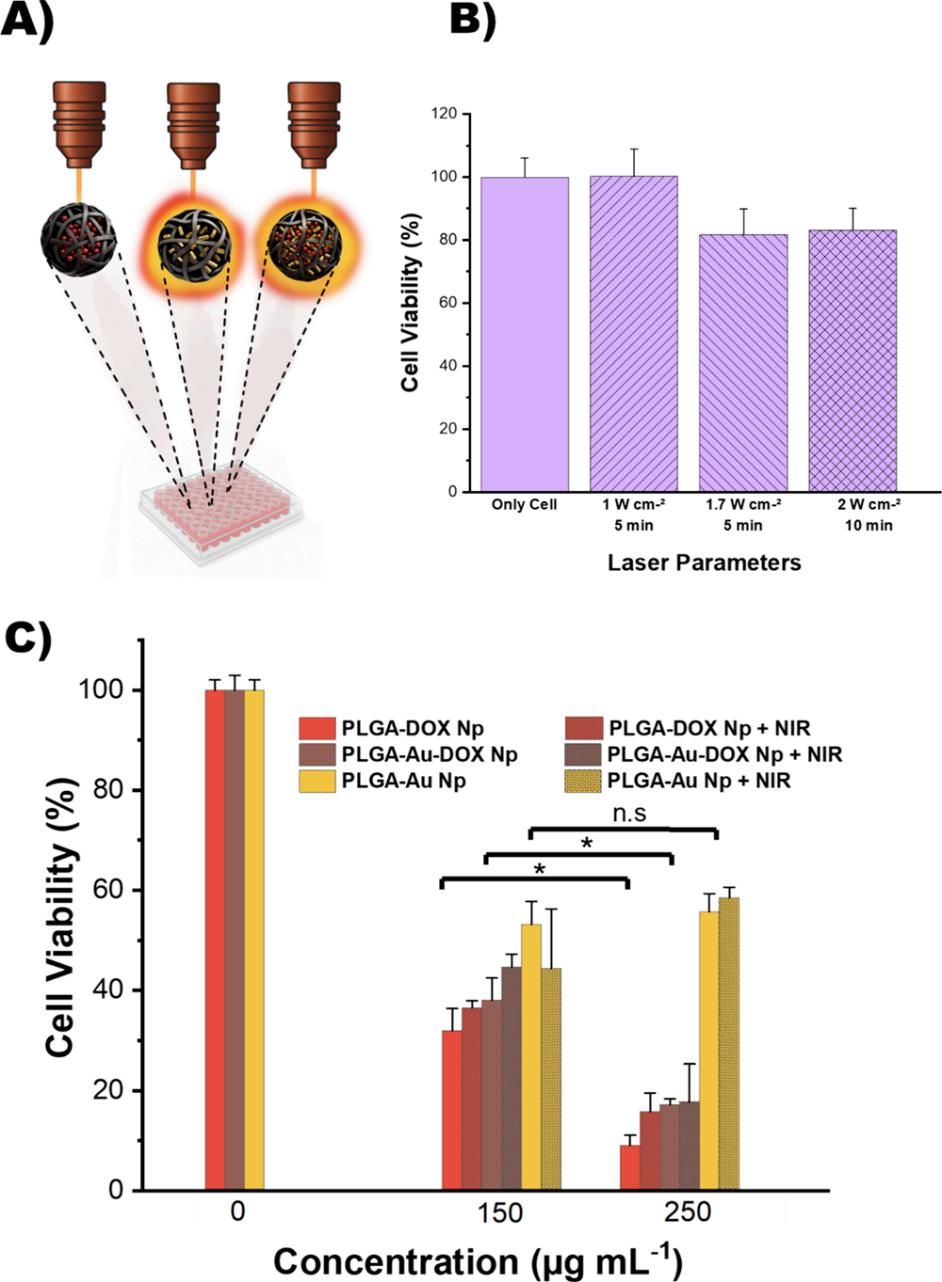
(A) Different nanoparticles in MCF-7 exposed to 808 nm continuous diode laser. (B) Cell viabilities of MCF-7 cells with the NIR application of different power densities and durations. (C) Cell viabilities of different concentrations of nanoparticles with and without NIR irradiation (1 W cm^–2^ for 5 min) (*n* = 3; *p ≤ 0.05, ns: not significant).

To apply the laser to cells containing nanoparticles, all treatment groups were seeded on the same 96-well plate to maintain uniform conditions between laser-exposed and nonexposed cells. PLGA-based nanoparticles (150 and 250 μg mL^–1^) were introduced following a 24-h attachment period. After 24 h of incubation, the wells were washed, and fresh medium was introduced. Certain wells were irradiated with an 808 nm laser (1 W cm^–2^, 300 s), whereas others remained under comparable ambient conditions without exposure. Cell viability was evaluated using the MTT test after an additional 24 h of incubation (Figure 7C). Here we have tested the performance of our structurally multimodal nanoparticles (dual Dox and AuNR loaded ones, PLGA-Au-Dox) comparing with the single cargo loaded ones (PLGA-Au, PLGA-Dox) that contain either Dox or AuNR at two different concentrations. To better assess the heating effects in cells, higher nanoparticle concentrations (150 and 250 μg mL^–1^) were employed, as these levels are more likely to induce detectable thermal responses.

When DOX was codelivered with gold nanorods (PLGA-Au-DOX), cell viability was 38 ± 4% without laser irradiation and 45 ± 3% with NIR (*p* > 0.05), indicating that the photothermal effect at 150 μg/mL is hindered likely due to the strong baseline cytotoxicity of DOX at this concentration, combined with potential NIR-induced alterations in drug release kinetics that may transiently reduce intracellular drug availability. Also, a previous report indicated that measurable photothermal benefits in MCF-7 cells appeared only above certain power densities, underscoring the role of power density in treatment efficacy.^1^

Additionally, NIR did not have a significant effect on cell viability of solely AuNR-loaded groups (PLGA-Au) where almost similar cell viability is obtained with and without NIR treatment (Figure 7C). This is in agreement with the concentration dependent data where increasing the concentration of the PLGA-Au from 150 to 250 μg mL^–1^ also did not lead to a significant change in the cell viability. This result supports that chemotherapy is the dominant driving factor for cell death, while the heat generated by the laser treatment is likely offset by PLGA, which may act as a nutrient source for cells. This claim is further supported by a study showing that 808 nm laser irradiation (2.5 W cm^–2^, 10 min) increased the viability of MCF-7 breast cancer cells treated with PLGA or folic acid-functionalized PLGA nanoparticles. The authors attributed this concentration-dependent increase to the natural origin of these materials.^36^ This supports the view that PLGA may promote cell growth and further highlighting its biocompatibility as a multimodal nanocarrier.

At 150 μg mL^–1^, PLGA-DOX exhibited 32 ± 4% cell viability, while PLGA-DOX + NIR showed 37 ± 1%, indicating a modest and statistically insignificant variation in cytotoxicity (*p* > 0.05). For PLGA-DOX, no additional effect of NIR irradiation on cell viability was observed, which is expected since these particles do not contain AuNRs and thus cannot produce photothermal heating. The inclusion of this condition served as a control to directly compare with AuNR-containing formulations.

Notably, when focusing solely on concentration at 250 μg mL^–1^, all DOX-containing groups showed significantly higher cytotoxicity than at 150 μg mL^–1^ (Figures 7C and S16) (*p* < 0.05). For instance, PLGA-DOX (9 ± 2%) and PLGA-Au-DOX (17 ± 1%) yielded lower cellular viabilities at 250 μg mL^–1^ compared to at 150 μg mL^–1^ with 32 ± 4% and 38 ± 4% viabilities, respectively. This concentration-dependent effect may stem from enhanced drug availability or uptake, amplifying cytotoxic stress, and underscores the chemotherapeutic potential of DOX-loaded PLGA.

These findings clearly demonstrate that, under the tested conditions, the chemotherapeutic potency of DOX-containing formulations dominated over any additional benefit from NIR irradiation. The observed increase in cytotoxicity with higher DOX concentrations confirms the strong chemotherapeutic effect. This finding highlights that successful integration of photothermal and chemotherapeutic approaches requires careful adjustment of laser parameters and nanoparticle design. These results also suggest that, beyond a certain threshold, the pronounced effect of chemotherapy may mask potential photothermal contributions. Future work may include evaluation across multiple cancer cell lines and fine-tuning of the AuNR LSPR to more closely match the 808 nm irradiation wavelength, with the aim of promoting absorption-dominated heating and reducing scattering losses, which may enhance the photothermal effect without increasing laser power. Such insights are valuable for planning combination therapies under physiologically relevant conditions and for guiding the development of more effective in vivo protocols.

## 4 Conclusions

Acid-terminated PLGA nanoparticles synthesized via the miniemulsion method demonstrated favorable physicochemical properties and stability, supporting their use as dual-loaded nanocarrier platforms. Although PLGA is a well-established polymer, its use in this study represents a meaningful advancement through the rational design of a PLGA-based nanocarrier coloaded with gold nanorods (as a photothermal agent) and doxorubicin (as a chemotherapeutic agent). In this hybrid system, PLGA not only acts as a biodegradable and biocompatible carrier but also enhances the stability of gold nanorods. Optimal AuNR to polymer ratios were identified, as excessive AuNR loading induced aggregation, while TGA confirmed successful encapsulation (∼48% efficiency). DOX was effectively coloaded with AuNRs, maintaining suitable size, PDI, and colloidal stability. In vitro release studies showed that DOX release remained effective following AuNR incorporation. Long-term stability tests revealed that PLGA encapsulation preserved AuNR structure and optical integrity for over 110 days under repeated centrifugal force. PLGA-Au-DOX nanoparticles exhibited efficient photothermal heating, with temperature increases up to 25 °C under NIR irradiation, highlighting its promise as a dual-loaded nanocarrier. Encapsulation of gold nanorods within the PLGA matrix provides a physical barrier between the cytotoxic CTAB layer on the surface of gold nanorods and biological environments, enabling the safe use of gold nanorods. Both PLGA and PLGA–Au NPs demonstrated good cytocompatibility in L929 mouse fibroblast cells, highlighting their potential as biocompatible and safe nanomaterials in biomedical applications. At the materials level, this study demonstrates the successful dual encapsulation of gold nanorods and doxorubicin within PLGA nanoparticles, supported by extensive physicochemical characterization and stability analyses. At the functional (biological) level, in vitro cytotoxicity in MCF-7 cells was primarily concentration-dependent, and chemotherapy-dominated with no significant synergy between NIR and DOX under tested conditions. Nevertheless, this study contributes to the literature by engineering a structurally multimodal nanocarrier through the dual-encapsulation strategy which is challenging and provides valuable information on the optimization of dual encapsulation and the long-term stability of such hybrid systems at the materials level.

Extended pH-dependent storage studies further highlighted the remarkable stability of purified PLGA nanoparticles for up to 342 days, emphasizing their potential for prolonged use under physiologically relevant environments. In the future, surface modification of these nanoparticles could be applied to enhance therapeutic targeting, while the successful development of a stable PLGA-based multimodal nanoplatform not only enables combined therapeutic applications but also opens opportunities for future imaging and diagnostic use, marking an important step toward multimodal nanomedicine.

## Supplementary Material



## Notes

Calibration curves for AuNR and DOX (Figures S1, S2A), drug release profiles (Figure S2B), FTIR spectra of PLGA before and after solvent evaporation (Figure S3), additional STEM images of PLGA–Au3 nanoparticles (Figure S4), FTIR and TGA analyses (Figures S5, S9), dry-state size analysis (Figure S6), SEM-EDS analysis of PLGA-Au and PLGA-Au-DOX (Figure S7), UV–vis spectra (Figure S8) and FTIR spectra (Figure S9) of nanoparticles, TGA graph of AuNR and dual encapsulated PLGA-Au-DOX Np (Figure S10), DSC analysis of PLGA polymer, PLGA Np, PLGA-Dox, PLGA-Au-Dox (Figure S11), colloidal stability data of AuNRs (Figure S12), AR analysis of AuNRs (Figure S13), long-term stability data of PLGA nanoparticles in different buffers (Figure S14, S15), and detailed p values obtained from Tukey’s post hoc test (Figure S16) (PDF)
