# Supplementary material for: Engineering Dual-Loaded PLGA Nanoparticles with Gold Nanorods and Doxorubicin as Robust Multimodal Nanoplatforms
Source: ACS Omega. 2026 Feb 6;11(6):10008–21. doi: 10.1021/acsomega.5c10824 (PMC12917820; doi:10.1021/acsomega.5c10824)
Supplement: Supplementary file 1 [file ao5c10824_si_001.pdf]

Supporting Information

Engineering Dual-Loaded PLGA Nanoparticles with Gold Nanorods and Doxorubicin as Robust Multimodal Nanoplatforms

*İrem S. İlçi[1], Yağmur Zengin[1], Banu Iyisan[1,2]**

[1]Biofunctional Nanomaterials Design (BIND) Laboratory, Institute of Biomedical Engineering, Bogazici University, 34684 Istanbul, Turkey

[2]Center for Targeted Therapy Technologies (CT3), Bogazici University, 34684, Istanbul, Turkey

*Corresponding author. E-mail: banu.iyisan@bogazici.edu.tr

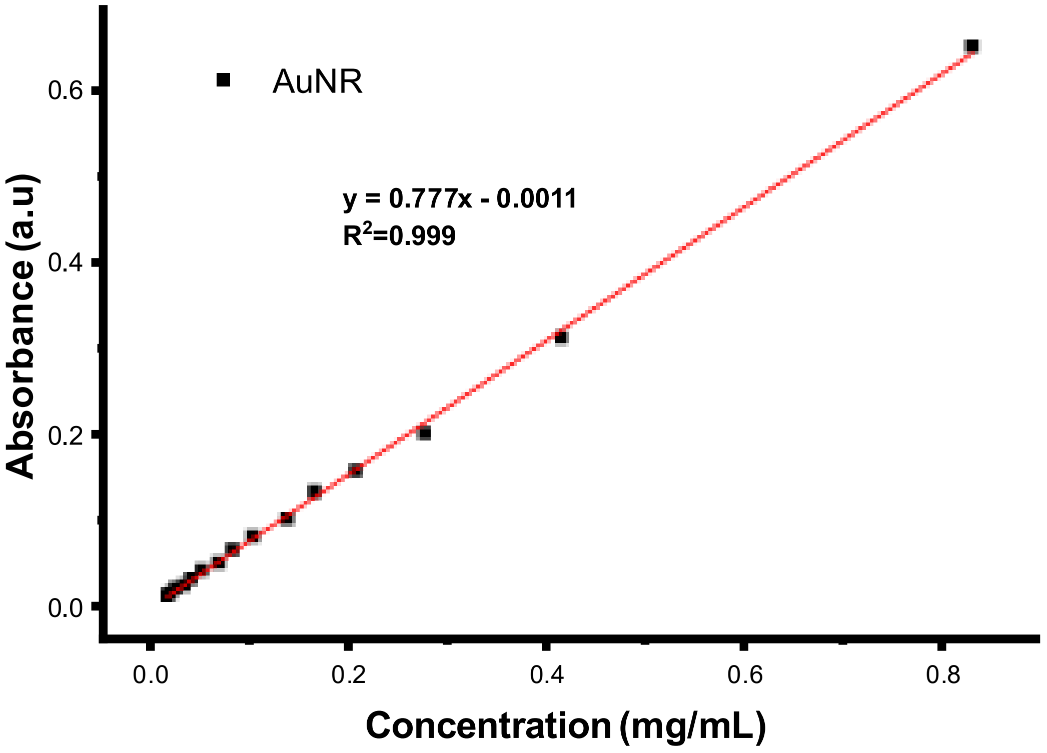

Figure S1. Calibration curve of gold nanorod (AuNR) in water measured at a wavelength of 808 nm.

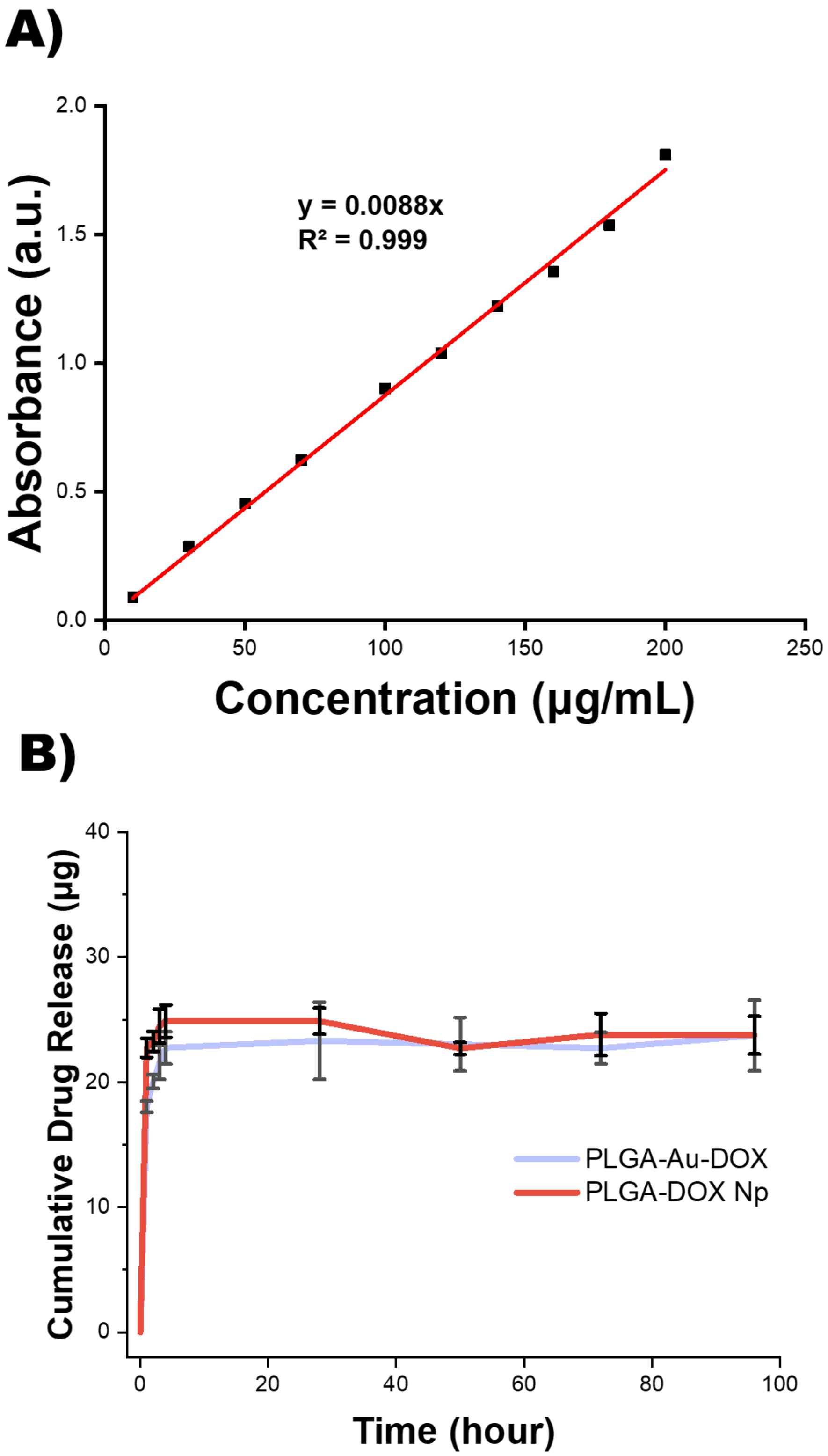

Figure S2. **(A)** Calibration curve of doxorubicin in DPBS**.** **(B)** Cumulative drug release profiles of PLGA-DOX and PLGA-Au-DOX nanoparticles in DPBS in pH 7.4 at 37°C.

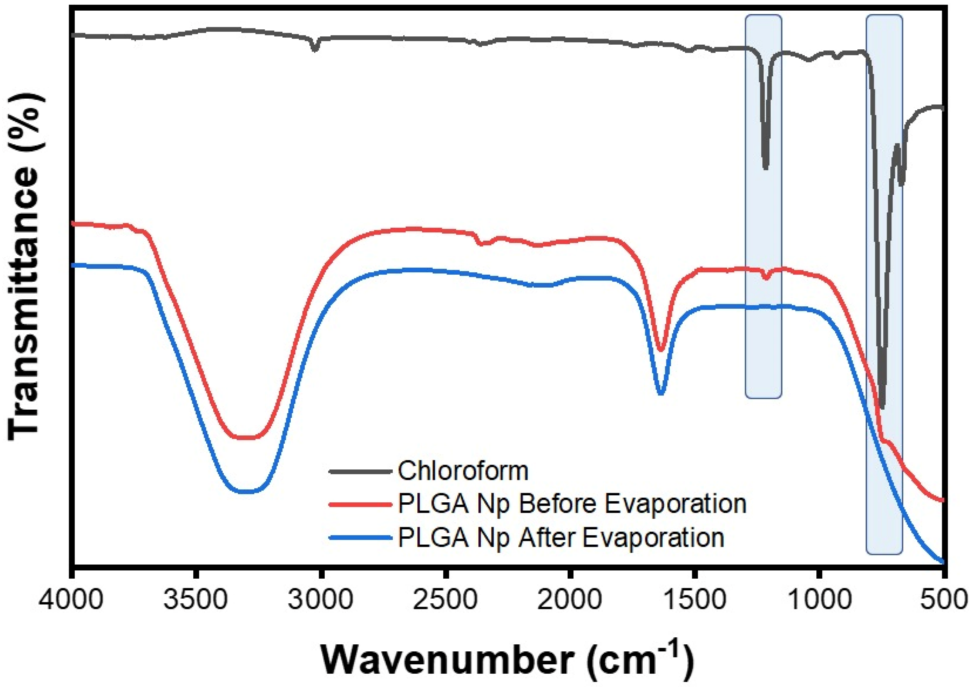

Figure S3. Fourier Transform Infrared Spectroscopy (FTIR) spectra of chloroform, PLGA nanoparticles (PLGA Np) before and after solvent evaporation

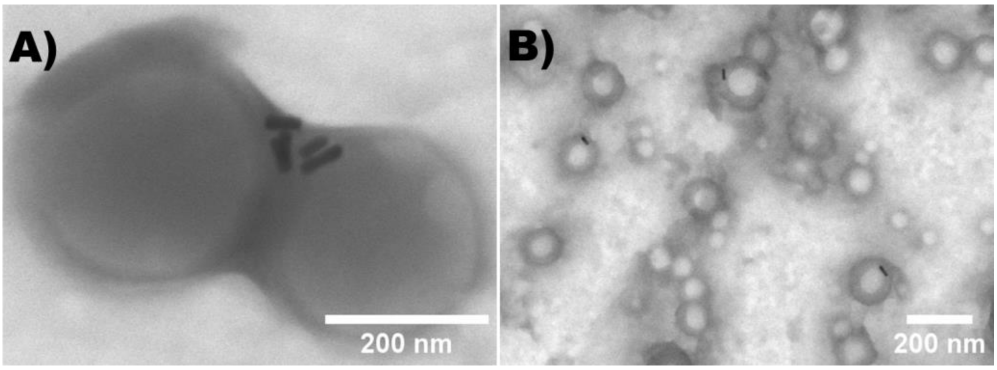

Figure S4. Scanning Transmission electron microscopy (STEM) images of PLGA-Au3 nanoparticles before purification: **(A)** and **(B)** represent different fields of the same formulation, showing spherical PLGA structures containing embedded AuNRs.

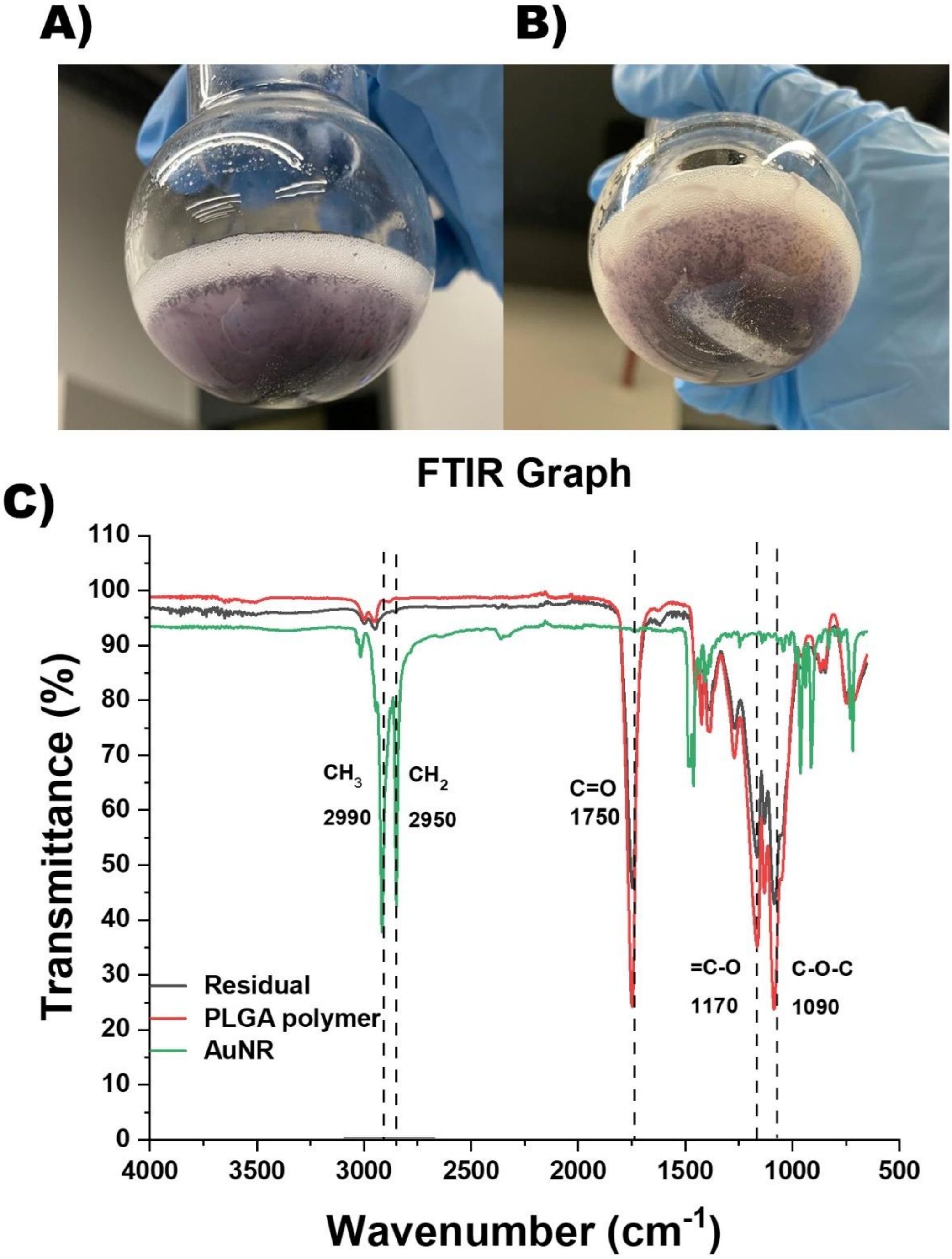

Figure S5. (A-B) Residues precipitated in the synthesis of the PLGA-Au5 encoded sample loaded with gold nanorods and **(C)** the FTIR graph of these residues, PLGA and AuNR.

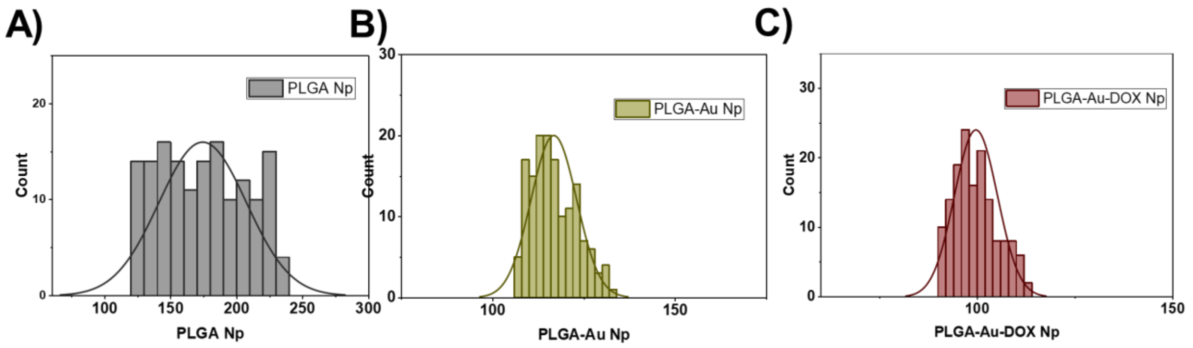

Figure S6. Dry-state particle diameter distributions obtained from STEM image analysis (n = 150).

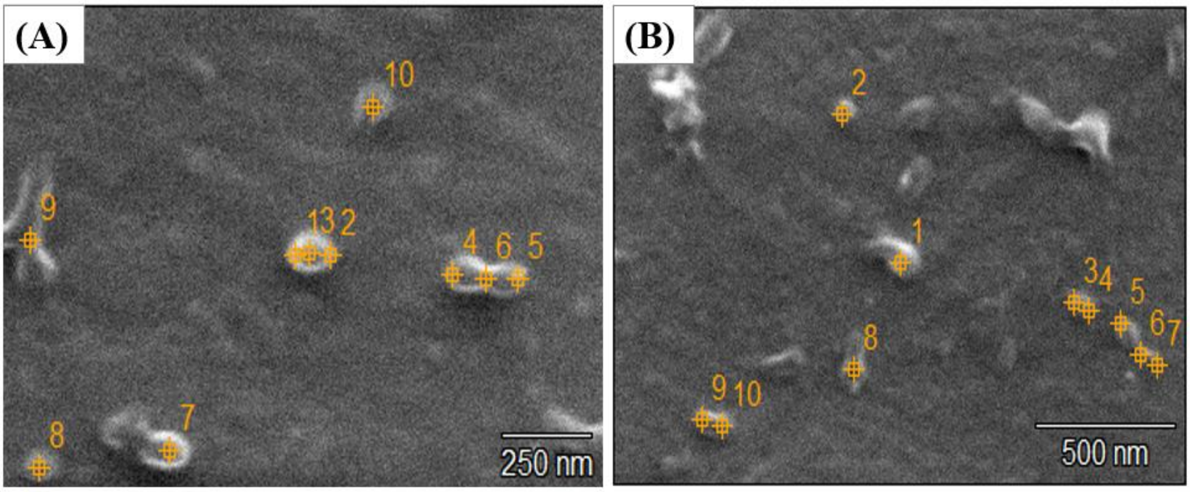

PLGA-Au	C	O	Au
**Point 1**	83.351	16.586	0.063
**Point 2**	82.338	16.980	0.682
**Point 3**	79.686	19.726	0.588
**Point 4**	82.326	16.065	1.609
**Point 5**	84.786	14.620	0.594
**Point 6**	81.217	18.103	0.680
**Point 7**	85.225	14.005	0.770
**Point 8**	86.392	13.608	0.000
**Point 9**	86.140	13.860	0.000
**Point 10**	84.386	13.358	2.256

PLGA-Au-DOX	C	O	Au
**Point 1**	73.137	26.177	0.686
**Point 2**	75.901	24.099	0.000
**Point 3**	77.100	20.963	1.937
**Point 4**	80.248	19.752	0.000
**Point 5**	79.474	20.163	0.000
**Point 6**	57.491	21.843	1.370
**Point 7**	79.640	20.018	0.341
**Point 8**	75.317	24.683	0.000
**Point 9**	74.920	21.318	3.762
**Point 10**	75.199	20.483	4.318

Figure S7. Scanning Electron Microscopy-Energy Dispersive Spectroscopy (SEM-EDS) analysis of **(A)** gold nanorod loaded PLGA nanoparticles (PLGA-Au) **(B)** gold nanorod and doxorubicin loaded PLGA nanoparticles (PLGA-Au-DOX)

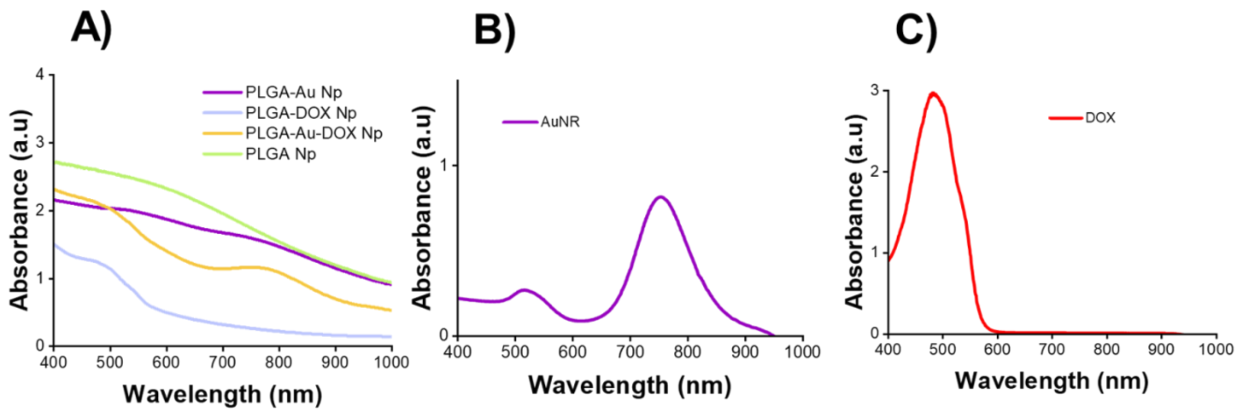

Figure S8. (A) UV-Vis absorbance spectra of free and loaded nanoparticles, **(B)** AuNR (0.714 mg mL^{-1}), and **(C)** doxorubicin (0.5 mg mL^{-1}).

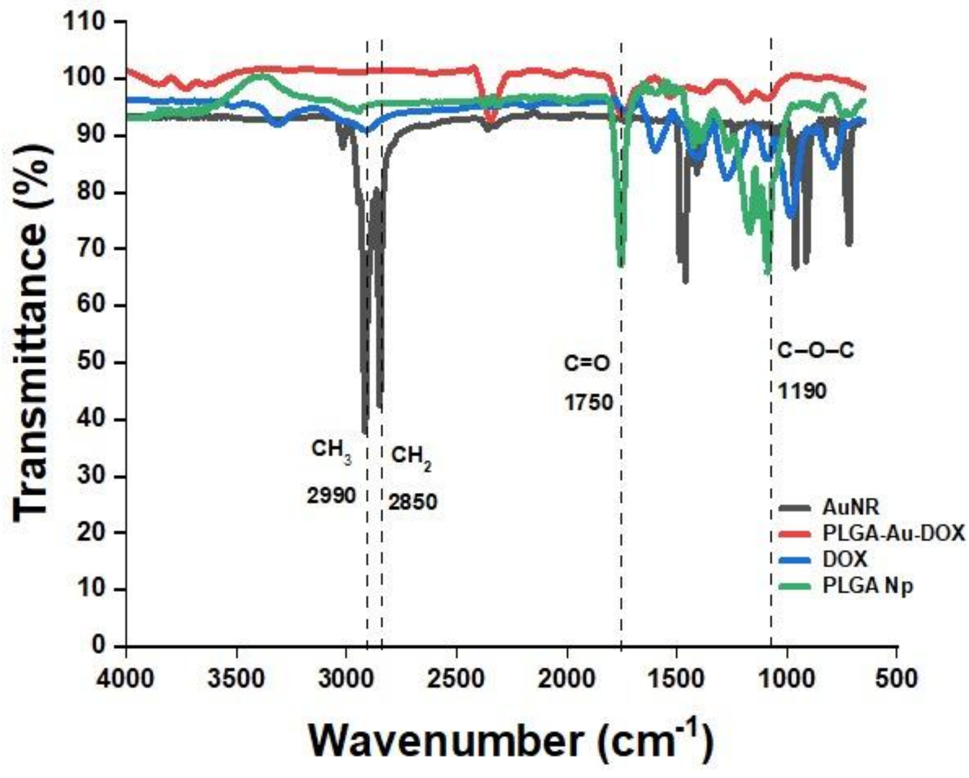

Figure S9. FTIR spectra of blank PLGA NPs, AuNRs, PLGA-Au-DOX NPs and free DOX.

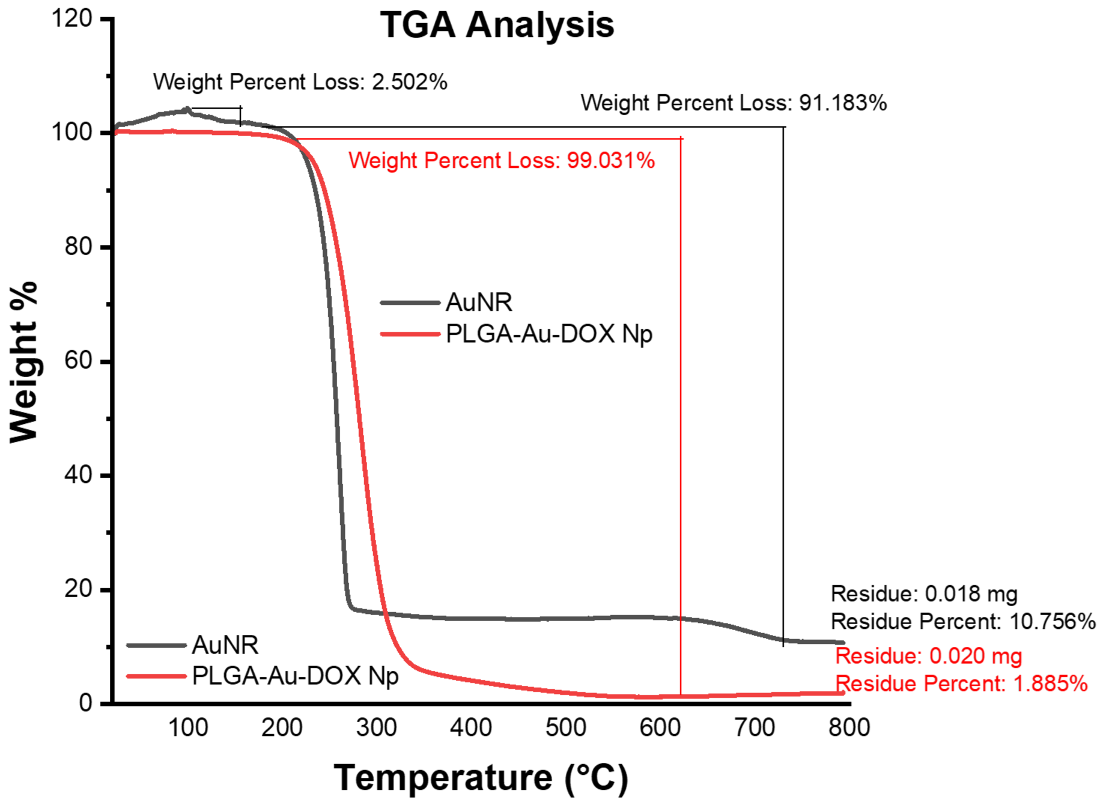

Figure S10. TGA analysis of the samples of PLGA-Au-DOX Np and AuNR.

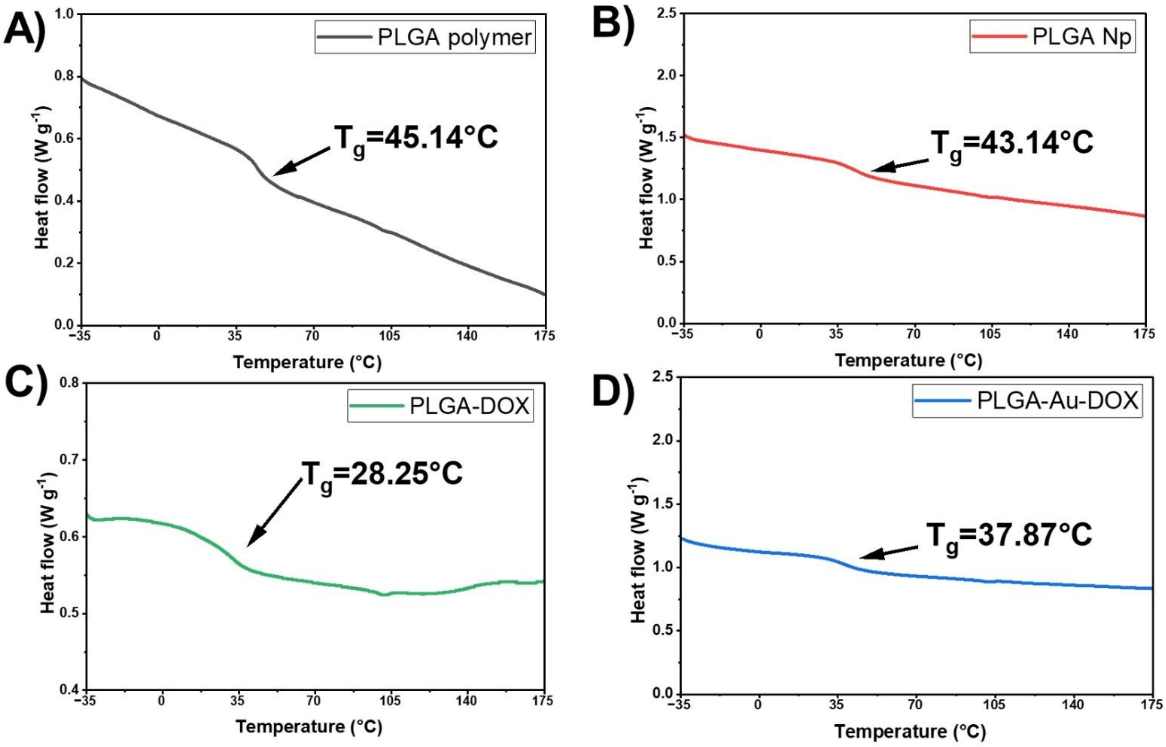

Figure S11. DSC analysis of PLGA polymer, PLGA nanoparticles (PLGA Np), doxorubicin loaded PLGA Np (PLGA-DOX Np), AuNR and doxorubicin loaded PLGA Nps (PLGA-Au-DOX Np)

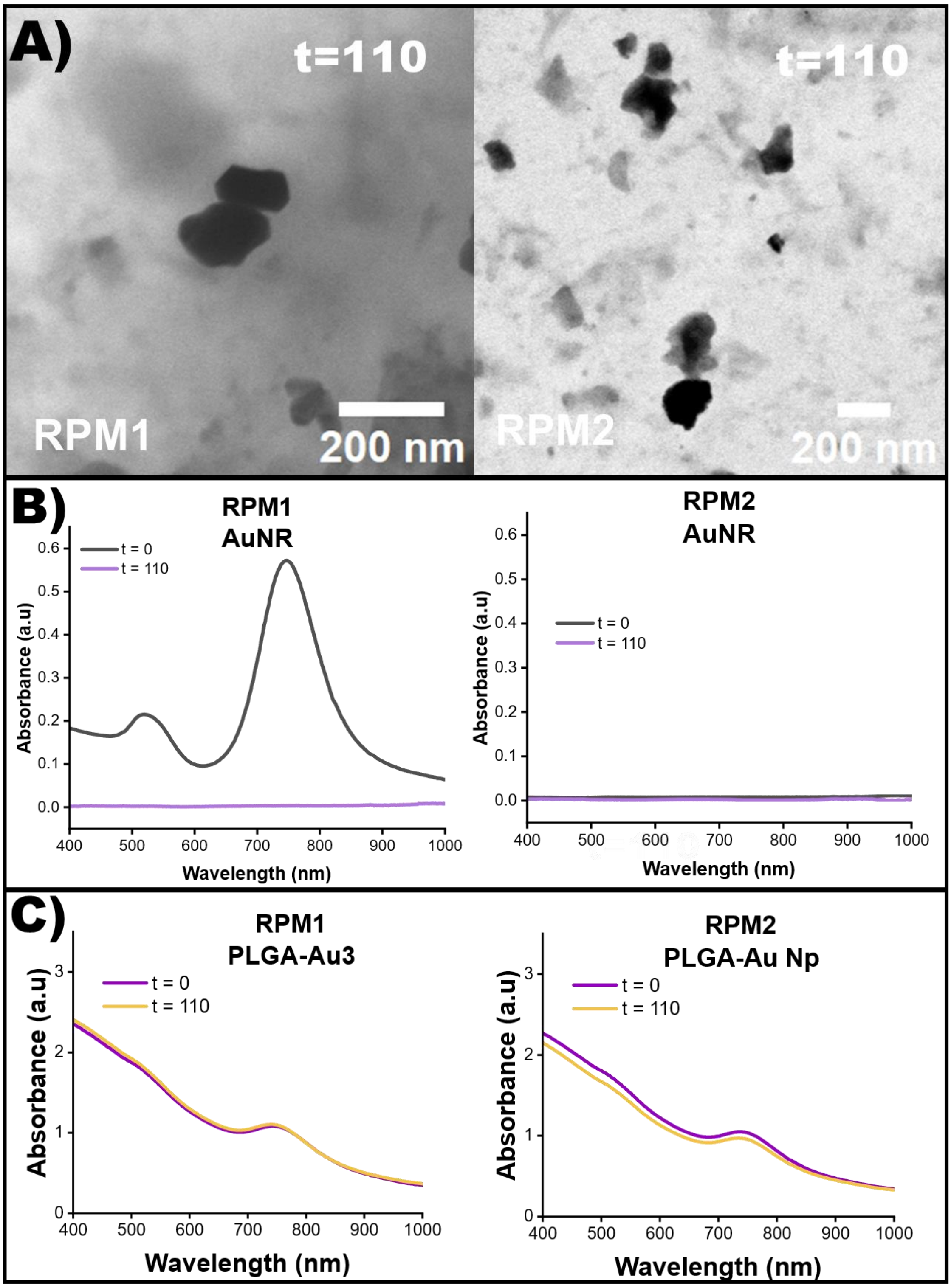

Figure S12. Colloidal stability of AuNRs. **(A)** STEM images of RPM1 and RPM2 over 110 days, **(B)** UV-Vis measurements of bare AuNRs under the different mechanical stress and **(C)** full spectra of PLGA-Au Np over 110 days.

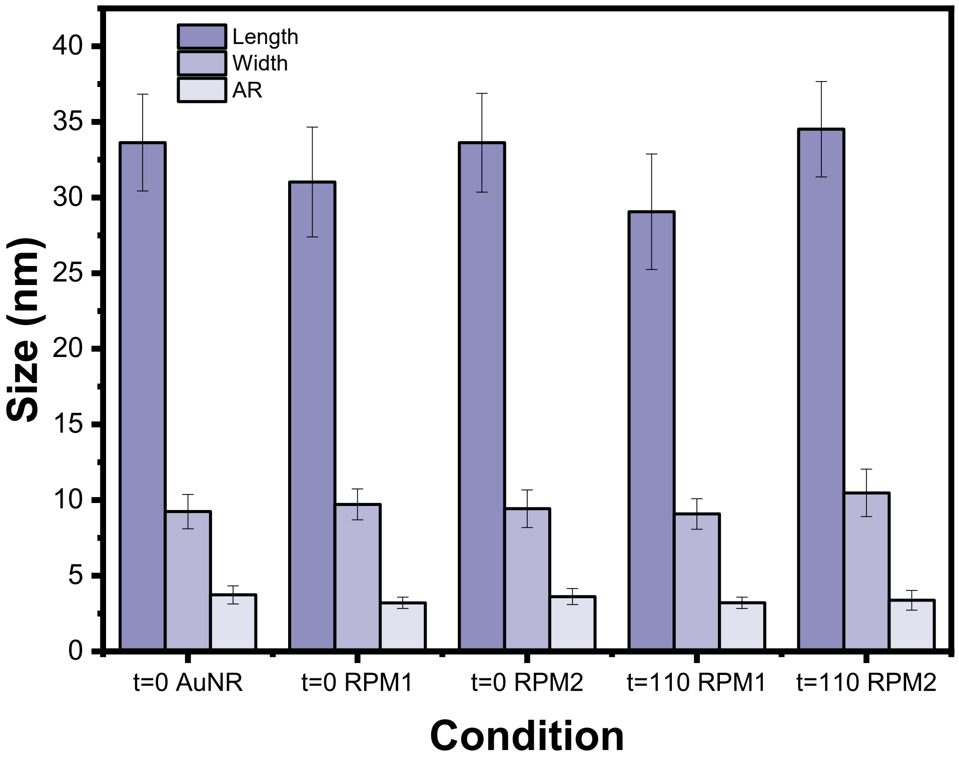

Figure S13. Length, width and aspect ratio (AR) of gold nanorods (AuNR) at day 0, before encapsulation (t=0, AuNR, RPM1) and AuNR encapsulated into PLGA Nps at day 0 (t=0) and day 110 (t=110) under different centrifugation conditions, RPM1 and RPM2 (n=20)

Table S1. Length, width, and aspect ratio (AR) of gold nanorods (AuNR) at day 0 before encapsulation (t=0, AuNR, RPM1) and AuNR encapsulated into PLGA Np at day 0 (t=0) and day 110 (t=110) under different centrifugation conditions, RPM1 and RPM2 (n=20).

Condition	Length (nm)	Width (nm)	AR
t=0 AuNR	33.6 ± 3.1	9.2 ±1.1	3.7 ± 0.5
t=0 / RPM1	31.0 ± 3.6	9.7 ± 1.0	3.2 ± 0.3
t=0 / RPM2	33.6 ± 3.2	9.4 ± 1.2	3.6 ± 0.5
t=110 / RPM1	29.1 ± 3.8	9.1 ± 1.0	3.2 ± 0.3
t=110 / RPM2	34.5 ± 3.1	10.5 ± 1.5	3.3 ± 0.6

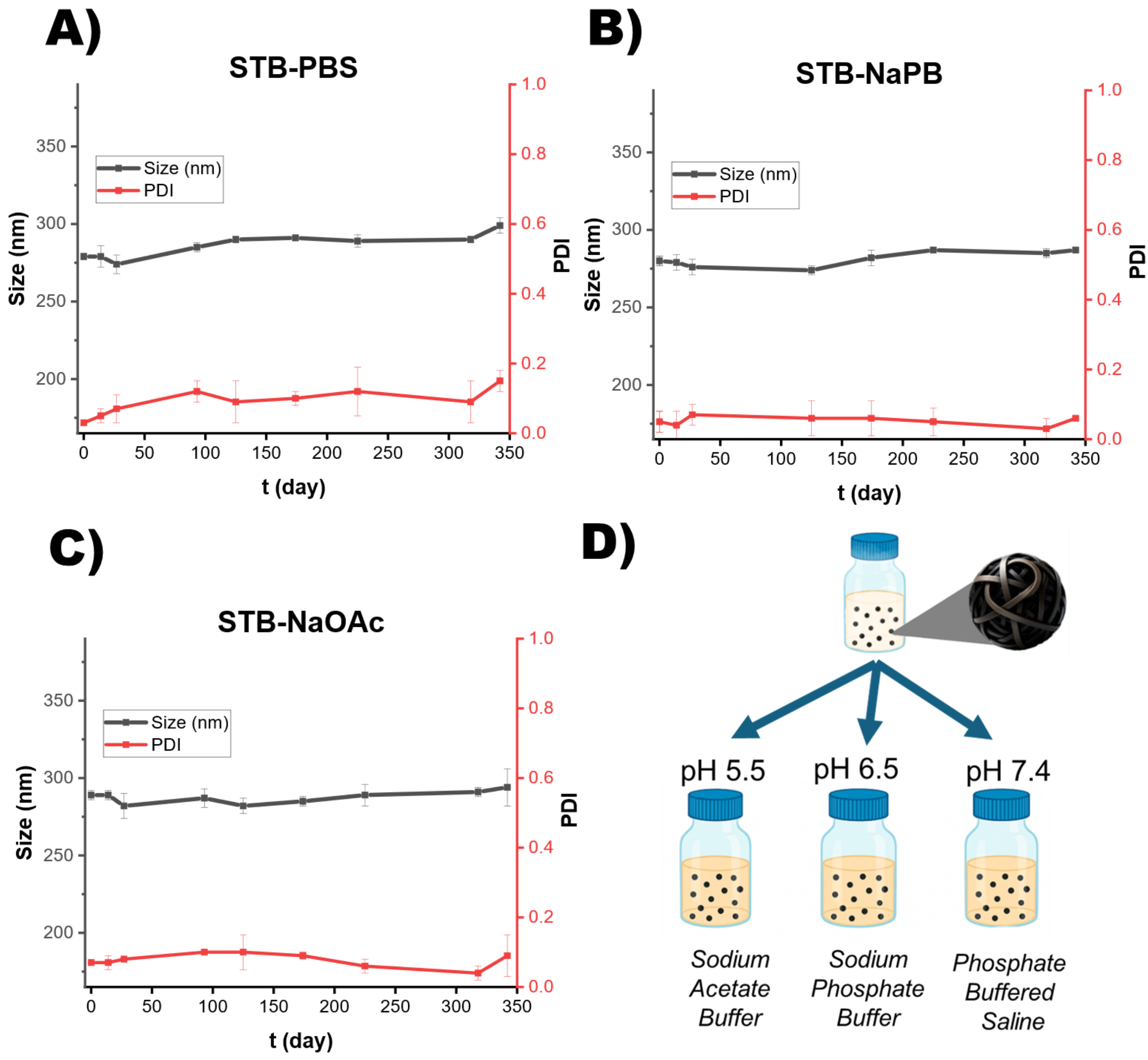

Figure S14. Stability results from the Stb-Buffer study, including almost one year of size and PDI data **(A)** in PBS, **(B)** in sodium phosphate, and **(C)** in sodium acetate. **(D)** Schematic representation of experimental design.

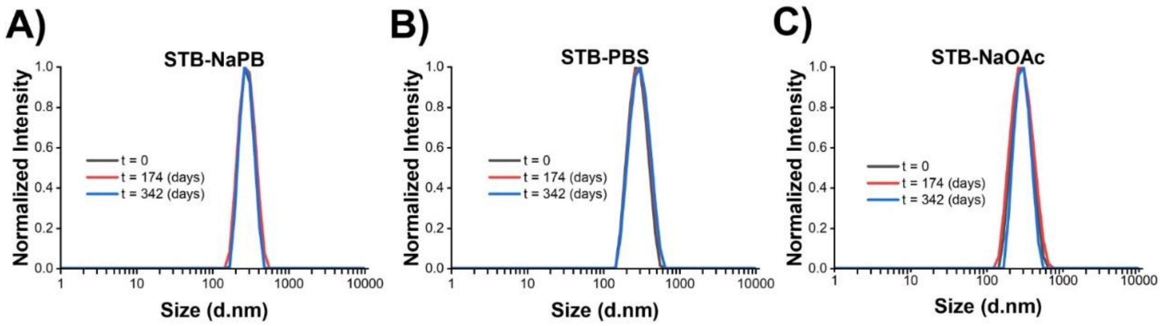

Figure S15. DLS intensity distribution curves of PLGA nanoparticles at t = 0, t = 174 and t = 342 days, **(A)** STB-NaPB, **(B)** STB-PBS, **(C)** STB-NaOAc, collected during the long-term stability study.

A)

Sample	P Values			
	150 μg mL^{-1}		250 μg mL^{-1}	
	NIR (-) vs NIR (+)		NIR (-) vs NIR (+)	
PLGA-Au-DOX Np	0.77063	ns	1	ns
PLGA-Au Np	0.32609	ns	0.99996	ns
PLGA-DOX Np	0.98349	ns	0.73934	ns

B)

Sample	P Values			
	NIR (+)		NIR (-)	
	150 μg mL^{-1} vs 250 μg mL^{-1}		150 μg mL^{-1}vs 250 μg mL^{-1}	
PLGA-Au-DOX Np	<0.0001	*	<0.0001	*
PLGA-Au Np	0.00826	*	0.99998	ns
PLGA-DOX Np	<0.0001	*	<0.0001	*

Figure S16. p-values obtained from Tukey's post hoc test (three-way ANOVA) for cell viability after treatment with PLGA-DOX, PLGA-Au-DOX, and PLGA-Au nanoparticles at **(A)** 150 and 250 μg mL^{-1} **(B)** under NIR– and NIR+ conditions on MCF-7 cell line (*$p \leq 0.05$; ns: not significant).
